# Supplementary material for: Effect of maternal cigarette smoking and alcohol consumption during pregnancy on birth weight and cardiometabolic risk factors in infants, children and adolescents: a systematic review protocol
Source: BMJ Open. 2022 Jul 14;12(7):e061811. doi: 10.1136/bmjopen-2022-061811 (PMC9295650; doi:10.1136/bmjopen-2022-061811)
Supplement: Supplementary data [file bmjopen-2022-061811supp001.pdf]

Supplementary file 1:

Table 1: SCOPUS search strategy

Search #	Search terms TITLE-ABS-KEY
#1	TITLE-ABS-KEY (maternal smoking)
#2	“tobacco smoke expos*”
#3	“smoking during pregnancy”
#4	“prenatal smok*”
#5	smoking pregnant
#6	“cigarette smoking”
#7	“maternal smok*”
#8	#1 OR #2 OR #3 OR #4 OR #5 OR #6 OR #7
#9	TITLE-ABS-KEY (alcohol exposure)
#10	maternal alcohol exposure
#11	maternal alcohol consumption
#12	fetal alcohol*
#13	alcohol exposure in utero
#14	ethanol
#15	ethanol exposure
#16	#9 OR #10 OR #11 OR #12 OR #13 OR #14 OR #15
#17	TITLE-ABS-KEY (birth outcomes)
#18	birth defects
#19	Low-Birth-Weight
#20	LBW
#21	intrauterine growth restriction
#22	IUGR
#23	“Fetal Alcohol Spectrum Disorders”
#24	FASD
#25	#17 OR #18 OR #19 OR #20 OR #21 OR #22 OR #23 OR #24
#26	TITLE-ABS-KEY (Metabolic syndrome)
#27	“Syndrome X”
#28	cardiovascular risk*
#29	lipid*
#30	Total cholesterol

#31	Triglycerides
#32	abnormal lipid profile
#33	dyslipidaemia
#34	Diabet*
#35	blood glucose
#36	Hyperglyc*
#37	metabolic outcomes
#38	obesity
#39	body composition
#40	Body Mass Index
#41	BMI
#42	waist circumference
#43	Body Weight
#44	skinfold
#45	anthropometry
#46	anthropometric measurements
#47	adiposity
#48	blood pressure
#49	hypertens*
#50	#26 OR #27 OR #28 OR #29 OR #30 OR #31 OR #32 OR #33 OR #34 OR #35 OR #36 OR #37 OR #38 OR #39 OR #40 OR #41 OR #42 OR #43 OR #44 OR #45 OR #46 OR #47 OR #48 OR #49
#51	TITLE-ABS-KEY (intima media thickness)
#52	carotid intima media thickness
#53	aortic intima media thickness
#54	vascular dysfunction
#55	atherosclero*
#56	vascular stiffness
#57	arterial stiffness
#58	#51 OR #52 OR #53 OR #54 OR #55 OR #56 OR #57
#59	(#8 AND #25) OR (#16 AND #25) OR (#8 AND #25 AND #50) OR (#16 AND #25 AND #50) OR (#16 AND #25 AND #58) OR (#8 AND #16 AND #25) OR (#8 AND #16 AND #50 AND #25) OR (#8 AND #16 AND #25 AND #58)
#60	#56 AND (LIMIT-TO (PUBYEAR , 2001-2022))

Table 2: Web of Science search strategy

Search #	**Search terms**
#1	(TS= ((maternal smoking) OR (“tobacco smoke expos*”) OR (“smoking during pregnancy”) OR (“prenatal smok*”) OR (smoke pregnant) OR (smoking pregnant) OR (“smoking during pregnancy”) OR (cigarette smoking) OR (“maternal smok*”)))
#2	(TS= ((alcohol exposure) OR (maternal alcohol exposure) OR (maternal alcohol consumption) OR (fetal alcohol) OR (foetal alcohol) OR (fetal alcohol exposure) OR (foetal exposure) OR (alcohol exposure in utero) OR (ethanol) OR (ethanol exposure) OR (“maternal alcohol expos*”)))
#3	(TS= ((birth outcomes) OR (birth defects) OR (Low-Birth-Weight) OR (LBW) OR (intrauterine growth restriction) OR (IUGR) OR (“Fetal Alcohol Spectrum Disorders”) OR (FASD)))
#4	(TS= ((Metabolic syndrome) OR (“Syndrome X”) OR (cardiovascular risk) OR (“cardiometabolic risk*”) OR (lipid*) OR (Total cholesterol) OR (Triglycerides) OR (abnormal lipid profile) OR (dyslipidemias) OR (dyslipidaemia) OR (diabetes) OR (diabetes mellitus) OR (blood glucose) OR (hyperglyc*) OR (metabolic outcomes) OR (obesity) OR (body composition) OR (Body Mass Index) OR (BMI) OR (waist circumference) OR (Body Weight) OR (skinfold) OR (anthropometry) OR (anthropometric measurements) OR (adiposity) OR (blood pressure) OR (hypertens*)))
#5	(TS= ((intima media thickness) OR (carotid intima media thickness) OR (aortic intima media thickness) OR (vascular dysfunction) OR (atherosclero*) OR (vascular stiffness) OR (arterial stiffness)))
#6	(#1 AND #3) OR (#2 AND #3) OR (#1 AND #3 AND #4) OR (#2 AND #3 AND #4) OR (#2 AND #5 AND #3) OR (#1 AND #2 AND #3) OR (#1 AND #2 AND #4 AND #3) OR (#1 AND #2 AND #5 AND #3)
#7	#6 AND (2001 OR 2002 OR 2003 OR 2004 OR 2005 OR 2006 OR 2007 OR 2008 OR 2009 OR 2010 OR 2011 OR 2012 OR 2013 OR 2014 OR 2015 OR 2016 OR 2017 OR 2018 OR 2019 OR 2020 OR 2021 OR 2022) PUBLICATION YEARS
#8	#7 AND Review Articles (Exclude – Document Types)
#9	#8 AND English (Languages)
