# Supplementary material for: Effect of maternal cigarette smoking and alcohol consumption during pregnancy on birth weight and cardiometabolic risk factors in infants, children and adolescents: a systematic review protocol
Source: BMJ Open. 2022 Jul 14;12(7):e061811. doi: 10.1136/bmjopen-2022-061811 (PMC9295650; doi:10.1136/bmjopen-2022-061811)
Supplement: Supplementary data [file bmjopen-2022-061811supp002.pdf]

APPENDIX A: DATA EXTRACTION FORM: Characteristics of studies

Authors	Year of publication	Country of data collection	Population age at outcome assessment	Sample size	Study design	Study setting	Data collection method

**Type of exposure**	**Source of data on exposure**	**Measure of the dependent variables**	**Birth/child health outcomes**	**Definition of outcome**	**Statistical methods used to measure outcome**	**Study findings**	**Conclusion**
