# Supplementary material for: Effect of maternal cigarette smoking and alcohol consumption during pregnancy on birth weight and cardiometabolic risk factors in infants, children and adolescents: a systematic review protocol
Source: BMJ Open. 2022 Jul 14;12(7):e061811. doi: 10.1136/bmjopen-2022-061811 (PMC9295650; doi:10.1136/bmjopen-2022-061811)
Supplement: Author's manuscript [file bmjopen-2022-061811.draft_revisions.pdf]

BMJ Open

BMJ Open is committed to open peer review. As part of this commitment we make the peer review history of every article we publish publicly available.

When an article is published we post the peer reviewers' comments and the authors' responses online. We also post the versions of the paper that were used during peer review. These are the versions that the peer review comments apply to.

The versions of the paper that follow are the versions that were submitted during the peer review process. They are not the versions of record or the final published versions. They should not be cited or distributed as the published version of this manuscript.

BMJ Open is an open access journal and the full, final, typeset and author-corrected version of record of the manuscript is available on our site with no access controls, subscription charges or pay-per-view fees (http://bmjopen.bmj.com).

If you have any questions on BMJ Open's open peer review process please email info.bmjopen@bmj.com

The effect of maternal smoking and alcohol consumption during pregnancy on birth weight and cardio-metabolic risk factors in children: A systematic review protocol

Journal:	*BMJ Open*
Manuscript ID	bmjopen-2022-061811
Article Type:	Protocol
Date Submitted by the Author:	07-Feb-2022

Complete List of Authors:	Hartel, Tammy; University of the Western Cape, Medical Bioscience<br>Turawa, Eunice; South African Medical Research Council, Burden of disease<br>De Smidt, Juley; University of the Western Cape, Medical Bioscience<br>Oelofse, Andre; University of the Western Cape, Medical Bioscience

Keywords:	Paediatric endocrinology < DIABETES & ENDOCRINOLOGY, Community child health < PAEDIATRICS, Maternal medicine < OBSTETRICS

SCHOLARONE™
Manuscripts

The effect of maternal smoking and alcohol consumption during pregnancy on birth weight and cardio-metabolic risk factors in children: A systematic review protocol

Tammy C Hartel*[1], Eunice B Turawa[2], Juléy JA De Smidt[1], André Oelofse[1]

[1]Department of Medical Bioscience, Faculty of Natural Sciences, University of the Western Cape, Private Bag X17, Bellville, 7530, South Africa.

[2]Burden of Disease Unit, South African Medical Research Council, PO Box 19070, Tygerberg, 7505, South Africa.

*Corresponding Author:

Tammy Hartel, Department of Medical Bioscience, Faculty of Natural Sciences, University of the Western Cape, Private Bag X17, Bellville, 7530, Cape Town, South Africa.

Tel: 082 778 1491

Email: 3366278@myuwc.ac.za

Word count: 2617

For peer review only

ABSTRACT

Introduction: Tobacco smoking and alcohol consumption during pregnancy is highly prevalent in low socioeconomic populations, which remains a public health concern, with adverse association between prenatal effects on birth outcomes and cardio-metabolic risk factors. However, the direct and indirect effects of prenatal smoking and alcohol consumption during pregnancy on cardio-metabolic risk in offspring have been rather inconsistent. Therefore, this review aims to provide a summary of up-to-date evidence-based birth outcomes and cardio-metabolic risk in children associated with alcohol and nicotine exposure *in utero*.

Methods and analysis: Relevant electronic databases: PubMed, Google scholar, Medline, Scopus and Web of Science will be searched to identify published articles from 2001– 2022. Using pre-specified eligible criteria, two reviewers, will independently select studies that meet the inclusion criteria. Quality assessment and risk of bias will be assessed using a risk of bias tool for observational studies, and data will be extracted for analysis. A meta-analysis will be performed to estimate pooled effect sizes if there is sufficient reporting and a low degree of heterogeneity. Sources of heterogeneity will be explored using subgroup analysis. The results of this review will be beneficial to researchers in the field of Developmental Origins of Health and Disease (DOHaD), health-care professionals, and policy- makers. The direct and indirect effects of alcohol and nicotine exposure on birth weight and cardio-metabolic outcomes in children and adolescents will be discussed. Research gaps in the literature will also be identified.

Ethics and Dissemination: Ethical clearance will not be required as this review will extract publicly available secondary data. The systematic review protocol was registered with the international prospective register of systematic reviews (PROSPERO): Registration number: CRD42021286630. The systematic review protocol will be included as a chapter in a doctoral thesis. In addition, findings from this review will be disseminated to peer-review publication.

Keywords: Cardio-metabolic risk factors, children, adolescents, alcohol exposure, maternal smoking, birth outcomes, systematic review.

Article Summary

Strengths and Limitations

- This study will only synthesise literature from primary human studies that investigate the association, correlation or causation between teratogen exposure and birth weight or cardio-metabolic risk factors in offspring and therefore, strengthens the evidence in this review.

For peer review only

- A comprehensive synthesis of all accessible data on outcomes of maternal smoking and alcohol consumption during pregnancy on birth weight and cardio-metabolic risk factors in children using a standardised risk of bias tool, while protocol adheres to Preferred Reporting Items for Systematic Reviews and Meta-Analyses (PRISMA) Protocols guidelines.
- The Grading of Recommendations assessment Development and Evaluation (GRADE) will be used to assess the quality and to strengthen the evidence from the review.
- Potential publication bias might limit the review and therefore, databases will be searched to find unpublished studies such as thesis dissertations and conference proceedings to minimise publication bias.
- Heterogeneity of included studies is expected and therefore may limit the generalizability of the study findings.

For peer review only

INTRODUCTION

The research field of the DOHaD have augmented in the past decade, with growing evidence of the detrimental effects of alcohol and nicotine exposure *in-utero* on the development of cardio-metabolic risk factors later in life [1,2]. Cardio-metabolic risk factors include increased central adiposity, elevated triglycerides, decreased high-density lipoprotein cholesterol (HDL-cholesterol), elevated blood pressure and hyperglycemia, which increases an individual's risk of developing type 2 diabetes mellitus and cardiovascular disease (CVD) [3]. Goodman, Armstrong and Magge, (2017) described Metabolic syndrome (MetS) as having at least three of these risk factors. Despite the challenges of defining metabolic syndrome in children and adolescents, Goodman, Armstrong and Magge (2017) emphasizes that clinical screening should rather shift the focus to cardio-metabolic risk factors to address the major risks associated with metabolic syndrome.

In addition, cardio-metabolic risk factors in offspring, specifically those exposed to nicotine or alcohol *in utero* have been reported previously [4–11]. The DOHaD may be driving the increasing prevalence of non-communicable diseases in developing countries as poverty, malnutrition and low birth weight are often prevalent in the same regions in developing countries such as South Africa [12]. In addition, exposure to undernutrition during pregnancy is often seen in low socioeconomic populations and may also be accountable for lower birth weight in offspring [12]. Equally important, infants born to mothers who smoked and consumed alcohol during pregnancy had the lowest birth weight compared to infants born to mothers who smoked or consumed alcohol only, or abstained [12]. Therefore, it is evident that tobacco and alcohol use during pregnancy results in adverse birth outcomes and influences cardiovascular health of the child during both childhood and adulthood. This is particularly important in a South African context where tobacco smoking and alcohol consumption during pregnancy is highly prevalent in low socioeconomic populations [12–16].

In summary, to prevent CVD later in life, it is important to assess adverse birth outcomes in infants and cardio-metabolic risk factors in early childhood, including dyslipidaemia, abdominal obesity, hypertension, high blood glucose and insulin resistance [17]. However, the evidence reported among studies investigating and reporting the relationship between exposure to these teratogens and cardio-metabolic outcomes in offspring are not consistent. Therefore, the direct and indirect effects of both maternal smoking and alcohol consumption during pregnancy on cardio-metabolic risk in offspring merits further investigation. The aim of this

For peer review only

review is to provide a summary of up-to-date evidence-based birth outcomes and cardio-metabolic outcomes in children associated with teratogen exposures *in utero*.

JUSTIFICATION FOR THIS REVIEW

Previous systematic reviews have addressed cardio-renal outcomes, body composition and metabolic outcomes in offspring exposed to alcohol during pregnancy [7,18]. Maternal smoking during pregnancy and child diabetes mellitus type 2, whereas only a few systematic reviews addressed maternal smoking during pregnancy and childhood overweight and obesity [17,19–21]. Prenatal smoking and cardio-metabolic risk factors were recently studied, but only among adults over 18 years of age [1]. Therefore, none of the latter have included all cardio-metabolic risk factors in offspring exposed to both maternal smoking and alcohol consumption during pregnancy. In addition, a review conducted in 2013 investigated maternal smoking during pregnancy and MetS in children over a decade ago and therefore is in need of updating [22]. Therefore, a systematic review will be conducted to summarize up-to-date evidence-based birth outcomes and cardio-metabolic outcomes in children and adolescents in association with teratogen exposures *in utero*.

AIMS, OBJECTIVES AND RESEARCH QUESTIONS

Aim

To provide a summary of up-to-date evidence-based birth outcomes and cardio-metabolic outcomes in children associated with teratogen exposures *in utero*.

Research question

What are the associated effects of smoking and alcohol consumption during pregnancy on birth weight, obesity, hypertension, diabetes, dyslipidaemia and vascular dysfunction?

Objectives

Primary objective:

To assess birth weight and cardio-metabolic risk factors associated with maternal smoking and alcohol consumption during pregnancy.

Secondary objectives:

For peer review only

i. Identify potential mediators in the development of cardio-metabolic risk factors and endothelial dysfunction in offspring exposed to alcohol and nicotine during pregnancy.
ii. Identify gaps in literature and provide recommendations for future clinical studies.

METHODS AND ANALYSIS

Protocol and Registration

The methods for this review was developed according to the Preferred Reporting Items for Systematic Reviews and Meta- Analyses protocols (PRISMA-P) and PRISMA statement [23,24]. The systematic review protocol was registered with the international prospective register of systematic reviews (PROSPERO): Registration number: CRD42021286630.

Criteria for Consideration of Studies

Types of Studies

Table 1 shows the list of inclusion and exclusion criteria. Types of studies will include human clinical studies that study the association, correlation or causation between maternal smoking and alcohol consumption and cardio-metabolic risk factors in their children. Observational studies: Prospective studies (cohort studies, birth cohort studies and prospective case-control studies) will be included. Data only from primary population-based studies will be included. Grey literature such as conference proceedings, ProQuest Dissertation and Theses database will be searched on the South African National Electronic Thesis and Dissertations (ETD) Portal and well as Google Scholar to reduce the risk of publication bias.

For peer review only

Table 1: Inclusion and exclusion criteria of studies.

Inclusion Criteria	Exclusion Criteria
• Studies that include pediatrics (Infants, children, adolescents).<br>• Observational studies: such as prospective cohort studies, birth cohort studies and prospective case-control studies will be included.<br>• Grey literature: Conference proceedings, PhD dissertations and unpublished articles.	• Studies that do not include infants, children or adolescents.<br>• Studies that do not include at least one outcome of interest will be excluded.<br>• Cross sectional studies, retrospective case-control studies, intervention studies, clinical trials, reviews and editorials, letters and abstracts.

- Studies investigating the association, correlation or causation between maternal smoking and/or alcohol consumption and cardiometabolic risk factors in offspring.
- Studies published after the year of 2001.

Types of Participants

Pediatric patients defined as:

i. Infants, children and adolescents between the ages of 0 years to 19 years old.
ii. Infants, children or adolescents exposed to maternal smoking only, maternal alcohol consumption only or both maternal smoking and alcohol during pregnancy.

Main Outcome Measures

i. Birth outcomes: Low birth weight.
ii. Anthropometry: Body Mass Index (BMI), overweight, Obesity, waist circumference.
iii. Blood pressure: Systolic blood pressure, Diastolic blood pressure, Hypertension, elevated blood pressure.
iv. Diabetes mellitus, Hyperglycemia.
v. Dyslipidaemia, Hypertriglyceridemia, High Low-density lipoprotein cholesterol, Low High-density lipoprotein cholesterol.
vi. Vascular dysfunction, increased intima-media thickness.

Search Methods for Identification of Studies

Using comprehensive search terms, relevant articles published in English between 2001 and 2022, and indexed in PubMed, Google scholar, Medline, Scopus and Web of Science will be identified for inclusion. The search strategies will incorporate both medical subject headings (MH) and free-text terms and will be adapted to suit each database using applicable controlled vocabulary. An experienced information specialist will review the search terms to provide inputs and to ensure that search terms are relevant and optimally sensitive to identify eligible studies. An example of the search strategy in PubMed is displayed in table 1, in which each of the terms for maternal smoke exposure or maternal alcohol exposure will be combined with each of the outcome terms described.

Electronic Searches

For peer review only

Relevant studies will be searched for and retrieved from five databases: PubMed, Google scholar, Medline, Scopus, Web of Science. In table 2, each of the terms for maternal smoke exposure or maternal alcohol exposure will be combined with each of the outcome terms described.

Table 2: Search strategy developed in PubMed.

Search #	Search terms
#1	maternal smoking [tiab] OR maternal smok* OR intrauterine tobacco smoke expos* [tiab] OR maternal smoking during pregnancy [tiab] OR prenatal smoking [tiab] OR prenatal smoke expos* [tiab] OR smoke pregnant [tiab] OR smoking pregnant [tiab] smoking during pregnancy [tiab] OR cigarette smoking [tiab]
#2	alcohol exposure [tiab] OR maternal alcohol exposure [tiab] OR maternal alcohol expos* OR maternal alcohol consumption [tiab] OR maternal alcohol drinking OR fetal alcohol [tiab] OR foetal alcohol [tiab] OR fetal alcohol exposure [tiab] OR foetal exposure [tiab] OR alcohol exposure in utero [tiab] OR ethanol [MH] OR ethanol exposure [tiab]
#3	birth outcomes [tiab] OR birth defects [tiab] OR infant, Low Birth Weight [MH] OR birth weight [MH] OR birth length [tiab] OR LBW [tiab] OR intrauterine growth restriction [tiab] OR IUGR [tiab] OR Fetal Alcohol Spectrum Disorders [MH] OR FASD [tiab] OR FASDs [tiab]
#4	Metabolic syndrome [MH] OR Syndrome X [tiab] OR cardiovascular risk [tiab] OR cardiometabolic risk* [tiab] OR lipid profile [tiab] OR Lipids [MH] OR Total cholesterol [tiab] OR Triglycerides [MH] OR abnormal lipid profile [tiab] OR dyslipidemias [MH], OR dyslipidaemia [tiab] OR diabetes [tiab] OR diabetes mellitus [MH] OR blood glucose [MH] OR hyperglycemia [MH] OR hyperglycaemia [tiab] OR metabolic outcomes [tiab] OR obesity [MH] OR body composition [MH] OR Body Mass Index [MH] OR BMI [tiab] OR waist circumference [MH] OR Body Weights and Measures [MH] OR skinfold [tiab] OR anthropometry [MH] OR anthropometric measurements [tiab] OR adiposity [tiab] OR blood pressure [tiab] OR hypertens* [tiab]
#5	intima media thickness [tiab] OR carotid intima media thickness [MH] OR aortic intima media thickness [tiab] OR vascular dysfunction [tiab] OR atherosclerosis [MH] OR atherosclerotic lesions [tiab] OR Plaque, Atherosclerotic [MH] OR vascular stiffness [MH] OR arterial stiffness [tiab]
#6	(#1 AND #3) OR (#2 AND #3) OR (#1 AND #3 AND #4) OR (#2 AND #3 AND #4) OR (#2 AND #5 AND #3) OR (#1 AND #2 AND #3) OR (#1 AND #2 AND #4 AND #3) OR (#1 AND #2 AND #5 AND #3)
#7	#6 NOT ("Animals"[Mesh] NOT "Humans"[Mesh])

For peer review only

#8	#7 AND "2001/01/01"[Date - Publication] : "2022/01/1"[Date - Publication]
#9	Systematic Review [Publication Type] OR "Review" [Publication Type]
#10	#8 NOT #9
#11	#10 AND (allchild[Filter])

Selection of Studies

The initial electronic database search will be performed to obtain all eligible records. Search results for each database will be exported to Mendeley, where duplicates will be removed, and the number noted. At least two reviewers, independently, will screen the titles and abstracts for potential eligible studies and to exclude irrelevant studies using the Rayyan intelligent systematic reviews (RIS) tool. Disagreement will be resolved by discussion and consensus between the two reviewers or through the third reviewer contribution. Eligibility criteria will be based on the PECO approach, an acronym representing the population, exposure, comparator and the outcome [25]. P (Population) of interest are infants, children and adolescents, the E (exposure) is maternal smoking, maternal alcohol consumption, or both, the C (comparator) is the control group with no exposure to smoking and alcohol during pregnancy, and the O (outcomes) of interest are birth weight and cardio-metabolic risk factors. Thereafter, the full text will be retrieved and exported to Mendeley and reviewed independently by two reviewers for a final selection of study into the review. Authors will be contacted if full texts cannot be retrieved. Experts in the field will be consulted to identify unpublished studies.

Studies will be selected if (ⅰ) relevant to the topic, (ⅱ) study design is epidemiological observational studies such as prospective cohort studies, birth cohort studies and case-control studies, (ⅲ) having maternal smoke exposure or maternal alcohol exposure, (ⅳ) having pregnant women as initially enrolled participants, (ⅴ) specified at least one adverse birth or child health outcome as an outcome of the investigation, (ⅵ) investigated the association, correlation or causation (ⅶ) having the largest sample size, if multiple studies used the same cohort. Only studies published in English will be included, but there will be no restriction regarding country, race, and gender. Studies focussing on mental health (including fetal alcohol syndrome), behavioural changes or cancer as the main outcome will be excluded. Any disagreement about the inclusion of studies will be resolved through consultation of a third reviewer.

In addition to the database search, reference lists of selected articles will be examined to identify relevant studies that have not been captured in the database search. The flow chart will be used to summarize the search and selection of eligible studies. The PRISMA flow chart template will be used to summarize the search and selection of eligible studies [24]. Reasons of any exclusion of studies will be documented.

Quality and Risk of Bias Assessment

Quality assessment and risk of bias will be measured using the Newcastle Ottawa scale (NOS) (http://www.ohri.ca/programs/clinical_epidemiology/oxford.asp) for cohort and case-control studies. Cohort and case-control studies will be awarded a maximum score of nine stars. High quality studies will be defined as a quality score of seven or above and a quality score below seven will be deemed low quality.

Data Extraction and Management

All data extracted from the selected studies will be captured into a Microsoft excel document. The following data will be extracted from each selected study: authors, date of publication, country of data collection, population age at outcome assessment, sample size, study design, study setting, data collection method, type of exposure, source of data on maternal smoking/alcohol consumption, measure of the dependent variables, birth or child health outcomes, definition of outcome, statistical methods used to measure outcome, adjusted covariates and effect estimates (Appendix A). Effect estimates will include relative risks (95% CI) or adjusted odds ratios (OR's (95% CI)). Findings will include all birth outcomes or cardio-metabolic outcomes tested for an association with teratogen exposures *in utero*. Thereafter, the data will be extracted and stored on Review Manager (Revman Cochrane) for analyses and reporting. PRISMA guidelines will be used to summarize and report the review findings [24].

Data Synthesis and Analysis

Data Analysis

Findings from each study will be reported as a risk ratio or an odd ratio with the 95% confidence interval for dichotomous variables or mean difference with the standard deviation for continuous variables. Studies with similar types of outcomes will be grouped to obtain feasible results on an overall estimate of effect. Due to the anticipated heterogeneity in included studies, a random effects meta-analysis will be considered.

For peer review only

Assessment of Heterogeneity

Heterogeneity will be evaluated through visual inspection of forest plots to judge the extent of Confidence Interval (CI) overlap. In addition, heterogeneity will be tested by the I^2 test. The I^2 statistic will classify heterogeneity. Heterogeneity among studies is expected due to risk factors defined differently according to age in children and adolescents. Therefore, the methods used to measure each health outcome are suspected to be different in children compared to adolescents. Additionally, heterogeneity may be due to characteristics such as gender, maternal health, maternal BMI and socioeconomic status. If the data allows, subgroup analysis will be performed. Similar risk factors will be grouped for comparison across studies. Risk factors according to age groups such as children or adolescents will be also be grouped.

Meta Analysis

Where there is sufficient reporting and a low degree of heterogeneity, a meta-analysis will be performed. Quantitative data will be pooled from individual studies to determine the pooled effect estimate with a fixed model preferred. A meta-analysis will be performed using Revman (Cochrane). After the meta-analysis, Grading of recommendations, assessment, development and evaluation (GRADEpro (Cochrane)) will be used to score and assess the overall certainty in the evidence for each outcome included in the analysis.

Subgroup Analysis

If the data allows, subgroup analysis will be performed. Similar risk factors will be grouped for comparison across studies. Risk factors according to age groups such as children or adolescents will also be grouped. The pooled effect estimate will not be determined if the source of heterogeneity cannot be explained through subgroup analysis. In addition, if a meta-analysis cannot be performed due to few articles, insufficient reporting of data or high degree of heterogeneity, a narrative synthesis will be reported.

Publication Bias

Begg's funnel chart will be used to perform a visual inspection and evaluation of publication bias on the selected data. This is to exclude any publication bias.

Patient and Public Involvement

It was not appropriate or possible to involve patients or the public in the design, or conduct, or reporting, or dissemination plans of our research.

For peer review only

Ethics and dissemination

The review will extract publicly available secondary data and therefore do not require ethical review. The study will be guided by experts with knowledge of content and methodology. The results will be written and submitted to a peer-reviewed journal. Results of the systematic review will be obtainable upon request through the University of the Western Cape's data repository (https://kikapu.uwc.ac.za/).

Protocol registration

The systematic review protocol was registered with the international prospective register of systematic reviews (PROSPERO): Registration number: CRD42021286630. Details of this protocol can be accessed on PROSPERO (https://www.crd.york.ac.uk/prospero/display_record.php?RecordID=286630).

FUNDING

This research was made possible through funding by the Council for Scientific and Industrial Research (CSIR) (Grant number: NA).

AUTHOR CONTRIBUTIONS

TCH, JJADS and AO conceptualised and developed the systematic review protocol. TCH wrote the first draft of the protocol. ET edited the manuscript and developed the search strategy. Both TCH and ET will independently screen and select the title and abstracts of articles. Thereafter, the full-text will be retrieved for a final review and selection. Any discrepancies regarding the eligibility of articles will be resolved through consensus and, in the event of a disagreement regarding the inclusion of articles, a consultation with a third reviewer will be held. Reasons for the exclusion of studies will be reported. TCH will extract, analyse and synthesis the data under critical guidance of ET. All authors will critically revise the successive drafts of the manuscript and approve the final version.

Conflict of Interest

The authors have no conflict of interest.

For peer review only

LIST OF ABBREVIATIONS

DOHaD	Developmental Origins of Health and Disease
CVD	Cardiovascular disease
MetS	Metabolic syndrome
BMI	Body Mass Index
SIDS	Sudden infant death syndrome
LBW	Low birth weight
IUGR	Intra-uterine growth restriction
SGA	Small-for-gestational age
HDL-cholesterol	High-density lipoprotein cholesterol
LDL cholesterol	Low-density lipoprotein cholesterol
cIMT	Carotid intima media thickness
aIMT	Aortic intima media thickness
PECO	Population, exposure, comparator, outcome
RIS tool	Rayyan intelligent systematic reviews tool
NOS	Newcastle Ottawa scale

For peer review only

REFERENCES

1 Kataria Y, Gaewsky L, Ellervik C. Prenatal smoking exposure and cardio-metabolic risk factors in adulthood : a general population study and a meta-analysis. *Int J Obes* 2018;**43**:763–73. doi:10.1038/s41366-018-0206-y

2 Leybovitz-Haleluya N, Wainstock T, Landau D, *et al.* Maternal smoking during pregnancy and the risk of pediatric cardiovascular diseases of the offspring: A population-based cohort study with up to 18-years of follow up. *Reprod Toxicol* 2018;**78**:69–74. doi:10.1016/j.reprotox.2018.03.009

3 Goodman E, Armstrong SC, Magge SN. The Metabolic Syndrome in Children and Adolescents : Shifting the Focus to Cardiometabolic Risk Factor Clustering. 2017;**140**.

4 Alfourti AMAB, Azzwali AAA, Azab AE. Effects of Maternal Nicotine Exposure during Pregnancy and Lactation on Blood Pressure of the Offspring and Blood Vessel Structure and Attenuation by Vitamin C. *East African Sch Journals Med Sci* 2019;**4421**.

5 Bakker H, Jaddoe VWV. Cardiovascular and metabolic influences of fetal smoke exposure. *Eur J Epidemiol* 2011;**26**:763–70. doi:10.1007/s10654-011-9621-2

6 Zhang CR, Kurniawan ND, Yamada L, *et al.* Early gestational ethanol exposure in mice: Effects on brain structure, energy metabolism and adiposity in adult offspring. *Alcohol* 2019;**75**:1–10. doi:10.1016/j.alcohol.2018.04.008

7 Akison LK, Reid N, Wyllie M, *et al.* Adverse health outcomes in offspring associated with fetal alcohol exposure: A systematic review of clinical and preclinical studies with a focus on metabolic and body composition outcomes. *Alcohol Clin Exp Res* 2019;**43**:1324–43. doi:10.1111/acer.14078

8 Weeks O, Fazeli PK, Goessling W, *et al.* Fetal alcohol spectrum disorder predisposes to metabolic abnormalities in adulthood Fetal alcohol spectrum disorder predisposes to metabolic abnormalities in adulthood. 2020;**130**:2252–69.

9 Molnar DS, Rancourt D, Schlauch R, *et al.* Tobacco Exposure and Conditional Weight-for-Length Gain by 2 Years of Age. *J Pediatr Psychol* 2017;**42**:679–88. doi:10.1093/jpepsy/jsw095

10 Oken E, Huh SY, Taveras EM, *et al.* Associations of maternal prenatal smoking with child adiposity and blood pressure. *Obes Res* 2005;**13**:2021–8. doi:10.1038/oby.2005.248

11 Cupul-Uicab LA, Skjaerven R, Haug K, *et al.* In utero exposure to maternal tobacco smoke and subsequent obesity, hypertension, and gestational diabetes among women in the MoBa cohort. *Environ Health Perspect* 2012;**120**:355–60. doi:10.1289/ehp.1103789

12 Mandy M, Nyirenda M. Developmental Origins of Health and Disease: The relevance to developing nations. *Int Health* 2018;**10**:66–70. doi:10.1093/inthealth/ihy006

13 Davoudi-Kiakalayeh A, Mohammadi R, Pourfathollah AA, *et al.* Alloimmunization in thalassemia patients: New insight for healthcare. *Int J Prev Med* 2017;**8**:1–5. doi:10.4103/ijpvm.IJPVM

14 Dukes K, Tripp T, Willinger M, *et al.* Drinking and smoking patterns during

For peer review only

pregnancy: Development of group-based trajectories in the Safe Passage Study. *Alcohol* 2017;**62**:49–60. doi:10.1016/j.alcohol.2017.03.001

15 Myers B, Koen N, Donald KA, *et al.* Effect of Hazardous Alcohol Use During Pregnancy on Growth Outcomes at Birth: Findings from a South African Cohort Study. *Alcohol Clin Exp Res* 2018;**42**:369–77. doi:10.1111/acer.13566

16 Peltzer K, Pengpid S. Maternal alcohol use during pregnancy in a general national population in South Africa. *South African J Psychiatry* 2019;**25**:1–5. doi:10.4102/sajpsychiatry.v25i0.1236

17 Cannon CP. Cardiovascular Disease and Modifiable Cardiometabolic Risk Factors. *Clin Cornerstone* 2007;**8**.

18 Al RET, Reid N, Akison LK, *et al.* Adverse Health Outcomes Associated With Fetal Alcohol Exposure : A Systematic Review Focused on Cardio – Renal Outcomes. 2010.

19 Oken E, Levitan EB, Gillman MW. Maternal smoking during pregnancy and child overweight : systematic review and meta-analysis. 2008;:201–10. doi:10.1038/sj.ijo.0803760

20 Rayfield S, Plugge E, Rayfield S, *et al.* Systematic review and meta-analysis of the association between maternal smoking in pregnancy and childhood overweight and obesity Stable URL : https://www.jstor.org/stable/26164426 Systematic review and meta-analysis of the association between maternal sm. 2016;**71**:162–73. doi:10.1136/jech-2016-207376

21 Sousa BA De, Horta BL. Maternal smoking during pregnancy and offspring body mass index and overweight : a systematic review and meta-analysis Tabagismo materno durante a gravidez e índice de massa corporal e excesso de peso entre os filhos : uma revisão sistemática e metanálise. 2019;**35**:1–18. doi:10.1590/0102-311X00176118

22 Seal N, Krakower G, Seal J. Maternal Smoking During Pregnancy and Metabolic Syndrome in Their Children. *TJNP J Nurse Pract* 2013;**9**:695–705. doi:10.1016/j.nurpra.2013.07.009

23 Petticrew M, Shekelle P, Stewart LA, *et al.* meta-analysis protocols (PRISMA-P) 2015 : elaboration. 2015;**7647**:1–25. doi:10.1136/bmj.g7647

24 Moher D, Liberati A, Tetzlaff J, *et al.* Preferred Reporting Items for Systematic Reviews and Meta-Analyses : The PRISMA Statement. 2009;**6**. doi:10.1371/journal.pmed.1000097

25 Wickham J, Stehman S, Gass L, *et al.* Identifying the PECO: A framework for formulating good questions to explore the association of environmental and other exposures with health outcomes. *Adv Ecol Res* 2019;**60**:1–24. doi:10.1016/j.envint.2018.07.015.Identifying

For peer review only

For peer review only

APPENDIX A: DATA EXTRACTION FORM: Characteristics of studies

Authors	Year of publication	Country of data collection	Population age at outcome assessment	Sample size	Study design	Study setting	Data collection method

For peer review only

Type of exposure	Source of data on exposure	Measure of the dependent variables	Birth/child health outcomes	Definition of outcome	Statistical methods used to measure outcome	Study findings	Conclusion

PRISMA 2020 Checklist

Section and Topic	Item #	Checklist item	Location where item is reported
**TITLE**			
Title	1	Identify the report as a systematic review.	
**ABSTRACT**			
Abstract	2	See the PRISMA 2020 for Abstracts checklist.	
**INTRODUCTION**			
Rationale	3	Describe the rationale for the review in the context of existing knowledge.	
Objectives	4	Provide an explicit statement of the objective(s) or question(s) the review addresses.	
**METHODS**			
Eligibility criteria	5	Specify the inclusion and exclusion criteria for the review and how studies were grouped for the syntheses.	
Information sources	6	Specify all databases, registers, websites, organisations, reference lists and other sources searched or consulted to identify studies. Specify the date when each source was last searched or consulted.	
Search strategy	7	Present the full search strategies for all databases, registers and websites, including any filters and limits used.	
Selection process	8	Specify the methods used to decide whether a study met the inclusion criteria of the review, including how many reviewers screened each record and each report retrieved, whether they worked independently, and if applicable, details of automation tools used in the process.	
Data collection process	9	Specify the methods used to collect data from reports, including how many reviewers collected data from each report, whether they worked independently, any processes for obtaining or confirming data from study investigators, and if applicable, details of automation tools used in the process.	
Data items	10a	List and define all outcomes for which data were sought. Specify whether all results that were compatible with each outcome domain in each study were sought (e.g. for all measures, time points, analyses), and if not, the methods used to decide which results to collect.	
	10b	List and define all other variables for which data were sought (e.g. participant and intervention characteristics, funding sources). Describe any assumptions made about any missing or unclear information.	
Study risk of bias assessment	11	Specify the methods used to assess risk of bias in the included studies, including details of the tool(s) used, how many reviewers assessed each study and whether they worked independently, and if applicable, details of automation tools used in the process.	
Effect measures	12	Specify for each outcome the effect measure(s) (e.g. risk ratio, mean difference) used in the synthesis or presentation of results.	
Synthesis methods	13a	Describe the processes used to decide which studies were eligible for each synthesis (e.g. tabulating the study intervention characteristics and comparing against the planned groups for each synthesis (item #5)).	
	13b	Describe any methods required to prepare the data for presentation or synthesis, such as handling of missing summary statistics, or data conversions.	
	13c	Describe any methods used to tabulate or visually display results of individual studies and syntheses.	
	13d	Describe any methods used to synthesize results and provide a rationale for the choice(s). If meta-analysis was performed, describe the model(s), method(s) to identify the presence and extent of statistical heterogeneity, and software package(s) used.	
	13e	Describe any methods used to explore possible causes of heterogeneity among study results (e.g. subgroup analysis, meta-regression).	
	13f	Describe any sensitivity analyses conducted to assess robustness of the synthesized results.	
Reporting bias assessment	14	Describe any methods used to assess risk of bias due to missing results in a synthesis (arising from reporting biases).	
Certainty assessment	15	Describe any methods used to assess certainty (or confidence) in the body of evidence for an outcome.	

For peer review only - http://bmjopen.bmj.com/site/about/guidelines.xhtml

PRISMA 2020 Checklist

Section and Topic	Item #	Checklist item	Location where item is reported
**RESULTS**			
Study selection	16a	Describe the results of the search and selection process, from the number of records identified in the search to the number of studies included in the review, ideally using a flow diagram.	
	16b	Cite studies that might appear to meet the inclusion criteria, but which were excluded, and explain why they were excluded.	
Study characteristics	17	Cite each included study and present its characteristics.	
Risk of bias in studies	18	Present assessments of risk of bias for each included study.	
Results of individual studies	19	For all outcomes, present, for each study: (a) summary statistics for each group (where appropriate) and (b) an effect estimate and its precision (e.g. confidence/credible interval), ideally using structured tables or plots.	
Results of syntheses	20a	For each synthesis, briefly summarise the characteristics and risk of bias among contributing studies.	
	20b	Present results of all statistical syntheses conducted. If meta-analysis was done, present for each the summary estimate and its precision (e.g. confidence/credible interval) and measures of statistical heterogeneity. If comparing groups, describe the direction of the effect.	
	20c	Present results of all investigations of possible causes of heterogeneity among study results.	
	20d	Present results of all sensitivity analyses conducted to assess the robustness of the synthesized results.	
Reporting biases	21	Present assessments of risk of bias due to missing results (arising from reporting biases) for each synthesis assessed.	
Certainty of evidence	22	Present assessments of certainty (or confidence) in the body of evidence for each outcome assessed.	
**DISCUSSION**			
Discussion	23a	Provide a general interpretation of the results in the context of other evidence.	
	23b	Discuss any limitations of the evidence included in the review.	
	23c	Discuss any limitations of the review processes used.	
	23d	Discuss implications of the results for practice, policy, and future research.	
**OTHER INFORMATION**			
Registration and protocol	24a	Provide registration information for the review, including register name and registration number, or state that the review was not registered.	
	24b	Indicate where the review protocol can be accessed, or state that a protocol was not prepared.	
	24c	Describe and explain any amendments to information provided at registration or in the protocol.	
Support	25	Describe sources of financial or non-financial support for the review, and the role of the funders or sponsors in the review.	
Competing interests	26	Declare any competing interests of review authors.	
Availability of data, code and other materials	27	Report which of the following are publicly available and where they can be found: template data collection forms; data extracted from included studies; data used for all analyses; analytic code; any other materials used in the review.	

From: Page MJ, McKenzie JE, Bossuyt PM, Boutron I, Hoffmann TC, Mulrow CD, et al. The PRISMA 2020 statement: an updated guideline for reporting systematic reviews. BMJ 2021;372:n71. doi: 10.1136/bmj.n71

For peer review only - http://bmjopen.bmj.com/site/about/guidelines.xhtml

For more information, visit: http://www.prisma-statement.org/

For peer review only

BMJ Open

The effect of maternal cigarette smoking and alcohol consumption during pregnancy on birth weight and cardio-metabolic risk factors in infants, children and adolescents: A systematic review protocol

Journal:	*BMJ Open*
Manuscript ID	bmjopen-2022-061811.R1
Article Type:	Protocol
Date Submitted by the Author:	17-May-2022
Complete List of Authors:	Hartel, Tammy; University of the Western Cape, Medical Bioscience<br>Turawa, Eunice; South African Medical Research Council, Burden of disease<br>Oelofse, Andre; University of the Western Cape, Medical Bioscience<br>De Smidt, Juley; University of the Western Cape, Medical Bioscience
**Primary Subject Heading**:	Paediatrics
Secondary Subject Heading:	Cardiovascular medicine, Research methods, Smoking and tobacco
Keywords:	Paediatric endocrinology < DIABETES & ENDOCRINOLOGY, Community child health < PAEDIATRICS, Maternal medicine < OBSTETRICS

SCHOLARONE™
Manuscripts

The effect of maternal cigarette smoking and alcohol consumption during pregnancy on birth weight and cardio-metabolic risk factors in infants, children and adolescents: A systematic review protocol

Tammy C Hartel*[1], Eunice B Turawa[2], André Oelofse[1], Juléy JA De Smidt[1].

[1]Department of Medical Bioscience, Faculty of Natural Sciences, University of the Western Cape, Private Bag X17, Bellville, 7530, South Africa.

[2]Burden of Disease Unit, South African Medical Research Council, PO Box 19070, Tygerberg, 7505, South Africa.

*Corresponding Author:

Tammy Hartel, Department of Medical Bioscience, Faculty of Natural Sciences, University of the Western Cape, Private Bag X17, Bellville, 7530, Cape Town, South Africa.

Tel: 082 778 1491

Email: 3366278@myuwc.ac.za

Authors:

Eunice B Turawa

Email: Eunice.Turawa@mrc.ac.za

André Oelofse

Email: aoelofse@uwc.ac.za

Juléy JA De Smidt

Email: jdesmidt@uwc.ac.za

Word count: 3076

For peer review only

ABSTRACT

Introduction: Tobacco smoking and alcohol consumption during pregnancy is highly prevalent in low socioeconomic populations, with adverse association between prenatal effects on birth outcomes and cardio-metabolic risk factors. However, the direct and indirect effects of prenatal cigarette smoking and alcohol consumption during pregnancy on cardio-metabolic risk in offspring have been rather inconsistent. This may be attributed to the amount and timing of exposure, influence of maternal, environmental and socioeconomic factors, or how risk factors may be defined. Therefore, this review aims to provide a summary of up-to-date evidence-based birth outcomes and cardio-metabolic risk in children associated with alcohol and tobacco exposure *in utero*.

Methods and analysis: Relevant electronic databases: PubMed, Scopus and Web of Science will be searched to identify published articles from January 1st, 2001 to review studies over a 20-year period. Clinical studies that investigate the association between maternal cigarette smoking or alcohol consumption and birthweight and cardio-metabolic risk factors in infants, children and adolescents will be included. Prospective cohort and case-control studies, and birth cohort studies will be included. Grey literature will be searched including conference proceedings, ProQuest Dissertation and Theses database and Google Scholar. Studies published in English will be included, with no restrictions regarding country, race and gender. Two reviewers, will independently select studies that meet the inclusion criteria. Eligibility criteria will be based on the population (infants, children, adolescents), exposure (maternal cigarette smoking, alcohol consumption, or both), comparator (control group with no exposure during pregnancy) and outcomes (birth weight and cardio-metabolic risk factors). Quality assessment and risk of bias will be assessed using a risk of bias tool for observational studies, and data will be extracted for analysis. A meta-analysis will be performed to estimate pooled effect sizes if there is sufficient reporting. Sources of heterogeneity will be explored using subgroup analysis.

Ethics and Dissemination: Ethical clearance will not be required as this review will extract publicly available secondary data. In addition, findings from this review will be disseminated via publication in a peer-review journal.

Registration: The systematic review protocol was registered with the international prospective register of systematic reviews (PROSPERO): Registration number: CRD42021286630.

Keywords: Cardio-metabolic risk factors, children, adolescents, alcohol exposure, maternal cigarette smoking, birth outcomes, systematic review.

For peer review only - http://bmjopen.bmj.com/site/about/guidelines.xhtml

Strengths and Limitations of this study

- This study will only synthesise literature from primary human studies that investigate the association, correlation or causation between alcohol or tobacco exposure during pregnancy and birth weight or cardio-metabolic risk factors in offspring, strengthening the evidence in this review.
- A comprehensive synthesis of all accessible data on outcomes of maternal cigarette smoking or alcohol consumption during pregnancy on birth weight and cardio-metabolic risk factors in children will make use of a standardised risk of bias tool.
- The Grading of Recommendations assessment Development and Evaluation (GRADE) will be used to assess the quality and to strengthen the evidence from the review.
- Potential publication bias might limit the review and therefore, databases will be searched to find unpublished studies such as thesis dissertations and conference proceedings to minimise publication bias.
- The review will gather evidence over an approximately two-decade period including studies from 2001 to date, which may limit the evidence.

For peer review only

For peer review only - http://bmjopen.bmj.com/site/about/guidelines.xhtml

INTRODUCTION

In 2020, the global prevalence of tobacco smoking was 6.5% among women [1]. This is attributed to a substantial decline in tobacco smoking of more than 40% in high socioeconomic countries over the past 50 years, however, there has been little to no decline in low to middle-income countries[1]. Globally, 32.4% of women over 16 years old are current alcohol consumers [2]. Despite declines in alcohol consumption and tobacco smoking, tobacco smoking remains a significant health burden, particularly among women of low socioeconomic status. These women are more likely to consume alcohol or use drugs (Cannabis, heroin, opioids, cocaine, methamphetamine) concurrently with tobacco smoking, and vice versa, during pregnancy [3–5]. To emphasise, in low socioeconomic populations, an alarming 61.2% of women consume alcohol, 56.3% smoke tobacco only and 37.4% consume alcohol only[4]. Women of low education level, low income, mental health disorders are more likely to smoke during pregnancy, relapse after pregnancy, and less likely to quit smoking, exacerbating the adverse effects on their offspring [6]. In addition, the prevalence of illicit drug use during pregnancy was 1.6% in 2020, with studies reporting a 7.4 times higher prevalence after toxicological analysis [3]. Similarly, biochemical analysis revealed that more than one in five pregnant women did not report their smoking status [7], resulting in under reported smoking and drinking patterns among pregnant women.

In the past decade, the research field of the Developmental Origins of Health and Disease (DOHaD) have augmented, with growing evidence of the detrimental effects of alcohol and tobacco exposure *in-utero* on the development of cardio-metabolic risk factors later in life [8,9]. Cardio-metabolic risk factors include increased central adiposity, elevated triglycerides, decreased high-density lipoprotein cholesterol (HDL-cholesterol), elevated blood pressure and hyperglycemia, which increases an individual's risk of developing type 2 diabetes mellitus and cardiovascular disease (CVD) [10]. Goodman, Armstrong and Magge, (2017) described Metabolic syndrome (MetS) as having at least three of these risk factors. Despite the challenges of defining metabolic syndrome in children and adolescents, Goodman, Armstrong and Magge (2017) emphasizes that clinical screening should rather shift the focus to cardio-metabolic risk factors to address the major risks associated with metabolic syndrome.

Moreover, cardio-metabolic risk factors in offspring, specifically those exposed to tobacco smoking or alcohol *in utero* have been reported previously [11–18]. Infants born to mothers who smoke, or those that consume alcohol during pregnancy, have an increased risk of adverse

For peer review only - http://bmjopen.bmj.com/site/about/guidelines.xhtml

birth outcomes such as low birth weight [7,19]. This is exacerbated in infants born to mothers who smoked tobacco and consumed alcohol during pregnancy compared to infants born to mothers who smoked only, consumed alcohol only, or abstained completely [20]. Therefore, birth weight is an important risk factor for cardiovascular disease, and is significantly associated with BMI, skinfold thickness and increased prevalence of obesity in adolescence [21,22]. Birth weight is also associated with dyslipidaemia, altered glucose metabolism, hypertension, as well as low- grade inflammation [23].

Whether infants are born small or large for gestational age, both increase their risk of overweight or obesity later in life due to catch-up growth [23–25]. Moreover, Koklu, Kurtoglu and Akcakuset et al (2006) reported a significantly higher aortic intima-media thickness, that was associated with hypertriglyceridemia in infants that were born small for gestational age [26]. Low birth weight as well as small for gestational age is also associated with higher carotid intima-media thickness values in children and linked to metabolic abnormalities, such as dyslipidemia, abdominal obesity, hypertension and development of insulin resistance [24,27,28]. Thus, overweight or obese children born with low birth weight are more likely to present with elevated systolic and diastolic blood pressures, triglycerides and low density lipoprotein cholesterol blood concentrations [29]. It is evident that tobacco smoking and alcohol use during pregnancy results in adverse birth outcomes which increases cardiometabolic health risks in both childhood and adulthood [30]. Therefore, the DOHaD may play a significant role in the development of cardiometabolic diseases in developing countries as poverty, malnutrition, licit and illicit drug use during pregnancy and low birth weight are often prevalent in the same regions [12].

In summary, to prevent CVD later in life, it is important to assess adverse birth outcomes in infants and cardio-metabolic risk factors in early childhood, including low birth weight, dyslipidaemia, abdominal obesity, hypertension, high blood glucose and insulin resistance [31]. This is particularly a concern in a South African context where tobacco smoking and alcohol consumption during pregnancy is highly prevalent in low socioeconomic populations [4,20,32–34]. However, the evidence reported among studies investigating and reporting the relationship between exposure to alcohol and tobacco during pregnancy and cardio-metabolic outcomes in offspring are not consistent. Therefore, the direct and indirect effects of maternal cigarette smoking or alcohol consumption during pregnancy on cardio-metabolic risk in offspring merits further investigation. The aim of this review is to provide a summary of up-

For peer review only

to-date evidence-based birth outcomes and cardio-metabolic outcomes in children associated with alcohol and tobacco exposure *in utero*.

JUSTIFICATION FOR THIS REVIEW

Previous systematic reviews have addressed cardio-renal outcomes, body composition and metabolic outcomes in offspring exposed to alcohol during pregnancy [14,35]. For example, maternal smoking during pregnancy and child diabetes mellitus type 2, whereas only a few systematic reviews addressed maternal smoking during pregnancy and childhood overweight and obesity [31,36–38]. Prenatal smoking and cardio-metabolic risk factors were recently studied, but only among adults over 18 years of age [8]. Therefore, none of the latter have included all cardio-metabolic risk factors in offspring and both maternal cigarette smoking or alcohol consumption during pregnancy. In addition, a review conducted in 2013 investigated maternal smoking during pregnancy and MetS in children and is in need of updating [39]. Therefore, a systematic review will be conducted to summarize up-to-date evidence-based birth outcomes and cardio-metabolic outcomes in children and adolescents in association with alcohol use or maternal cigarette smoking during pregnancy.

AIMS, OBJECTIVES AND RESEARCH QUESTIONS

Aim

To provide up-to-date evidence-based birth outcomes and cardio-metabolic outcomes in children associated with maternal cigarette smoking only, maternal alcohol consumption only or both maternal cigarette smoking and alcohol use during pregnancy.

Research question

What are the associated effects of cigarette smoking and alcohol consumption during pregnancy on birth weight, obesity, hypertension, diabetes, dyslipidaemia and vascular dysfunction?

Objectives

Primary objective:

To assess birth weight and cardio-metabolic risk factors associated with maternal cigarette smoking and alcohol consumption during pregnancy.

Secondary objectives:

For peer review only - http://bmjopen.bmj.com/site/about/guidelines.xhtml

i. Identify potential mediators in the development of cardio-metabolic risk factors and vascular dysfunction in offspring exposed to alcohol consumption and cigarette smoking during pregnancy.

ii. Identify gaps in literature and provide recommendations for future clinical studies.

METHODS AND ANALYSIS

Protocol and Registration

The methods for this review were developed according to the Preferred Reporting Items for Systematic Reviews and Meta- Analyses protocols (PRISMA-P) and PRISMA 2020 statement [40,41]. The systematic review protocol was registered with the international prospective register of systematic reviews (PROSPERO): Registration number: CRD42021286630.

Criteria for Consideration of Studies

Types of Studies

Table 1 shows the list of inclusion and exclusion criteria. Types of studies will include human clinical studies that study the association, correlation or causation between maternal cigarette smoking and alcohol consumption and cardio-metabolic risk factors in their children. Observational studies: Prospective studies (cohort studies, birth cohort studies and prospective case-control studies) will be included. Data only from primary population-based studies will be included. Grey literature such as conference proceedings, ProQuest Dissertation and Theses database will be searched on the South African National Electronic Thesis and Dissertations (ETD) Portal and well as Google Scholar to reduce the risk of publication bias.

Table 1: Inclusion and exclusion criteria of studies.

Inclusion Criteria	Exclusion Criteria
• Studies that include pediatrics (Infants, children, adolescents).<br>• Observational studies: such as prospective cohort studies, birth cohort studies and prospective case-control studies will be included.<br>• Grey literature: Conference proceedings, PhD dissertations and unpublished articles.<br>• Tobacco smoking in the form of cigarettes.	• Studies that do not include infants, children or adolescents.<br>• Studies that do not include at least one outcome of interest will be excluded.<br>• Cross sectional studies, retrospective case-control studies, intervention studies, clinical trials, reviews and editorials, letters and abstracts.

For peer review only - http://bmjopen.bmj.com/site/about/guidelines.xhtml

• Studies investigating the association, correlation or causation between maternal cigarette smoking and/or alcohol consumption and cardiometabolic risk factors in offspring.<br>• Studies published after the year 1st January, 2001.	• Other forms of tobacco smoking such as electronic cigarettes, cigars, bidis, waterpipe and "I quit original smoking" (IQOS) devices.

Types of Participants

Pediatric patients defined as:

i. Infants, children and adolescents between the ages of 0 years to 19 years old.
ii. Infants, children or adolescents exposed to maternal cigarette smoking only, maternal alcohol consumption only or both maternal cigarette smoking and alcohol during pregnancy.

Main Outcome Measures

i. Birth outcomes: Low birth weight, Birth weight.
ii. Anthropometry: Body Mass Index (BMI), Overweight, Obesity, Waist circumference.
iii. Blood pressure: Systolic blood pressure, Diastolic blood pressure, Hypertension, elevated blood pressure.
iv. Diabetes mellitus, Hyperglycemia.
v. Dyslipidaemia, Hypertriglyceridemia, High Low-density lipoprotein cholesterol, Low High-density lipoprotein cholesterol.
vi. Vascular dysfunction, increased intima-media thickness.

Search Methods for Identification of Studies

Using comprehensive search terms, relevant articles published in English from 2001, and indexed in PubMed, Google scholar, Scopus and Web of Science will be identified for inclusion. Relevant articles will be retrieved from 2001 to gather evidence over a two-decade period. The search strategies will incorporate both medical subject headings (MH) and free-text terms and will be adapted to suit each database using applicable controlled vocabulary [Supplementary file 1]. An experienced information specialist will review the search terms to provide inputs and to ensure that search terms are relevant and optimally sensitive to identify eligible studies. An example of the search strategy in PubMed is displayed in table 1, in which

For peer review only

For peer review only - http://bmjopen.bmj.com/site/about/guidelines.xhtml

each of the terms for exposure to maternal cigarette smoking or maternal alcohol consumption will be combined with each of the outcome terms described.

Electronic Searches

Relevant studies will be searched for and retrieved from three databases: PubMed, Scopus and Web of Science. In table 2, each of the terms for exposure to maternal cigarette smoking or alcohol consumption will be combined with each of the outcome terms described.

Table 2: Search strategy developed in PubMed.

Search #	**Search terms**
#1	maternal smoking [tiab] OR maternal smok* OR intrauterine tobacco smoke expos* [tiab] OR maternal smoking during pregnancy [tiab] OR prenatal smoking [tiab] OR prenatal smoke expos* [tiab] OR smoke pregnant [tiab] OR smoking pregnant [tiab] smoking during pregnancy [tiab] OR cigarette smoking [tiab]
#2	alcohol exposure [tiab] OR maternal alcohol exposure [tiab] OR maternal alcohol expos* OR maternal alcohol consumption [tiab] OR maternal alcohol drinking OR fetal alcohol [tiab] OR foetal alcohol [tiab] OR fetal alcohol exposure [tiab] OR foetal exposure [tiab] OR alcohol exposure in utero [tiab] OR ethanol [MH] OR ethanol exposure [tiab]
#3	birth outcomes [tiab] OR birth defects [tiab] OR infant, Low Birth Weight [MH] OR birth weight [MH] OR birth length [tiab] OR LBW [tiab] OR intrauterine growth restriction [tiab] OR IUGR [tiab] OR Fetal Alcohol Spectrum Disorders [MH] OR FASD [tiab] OR FASDs [tiab]
#4	Metabolic syndrome [MH] OR Syndrome X [tiab] OR cardiovascular risk [tiab] OR cardiometabolic risk* [tiab] OR lipid profile [tiab] OR Lipids [MH] OR Total cholesterol [tiab] OR Triglycerides [MH] OR abnormal lipid profile [tiab] OR dyslipidemias [MH], OR dyslipidaemia [tiab] OR diabetes [tiab] OR diabetes mellitus [MH] OR blood glucose [MH] OR hyperglycemia [MH] OR hyperglycaemia [tiab] OR metabolic outcomes [tiab] OR obesity [MH] OR body composition [MH] OR Body Mass Index [MH] OR BMI [tiab] OR waist circumference [MH] OR Body Weights and Measures [MH] OR skinfold [tiab] OR anthropometry [MH] OR anthropometric measurements [tiab] OR adiposity [tiab] OR blood pressure [tiab] OR hypertens* [tiab]
#5	intima media thickness [tiab] OR carotid intima media thickness [MH] OR aortic intima media thickness [tiab] OR vascular dysfunction [tiab] OR atherosclerosis [MH] OR atherosclerotic lesions [tiab] OR Plaque, Atherosclerotic [MH] OR vascular stiffness [MH] OR arterial stiffness [tiab]

For peer review only - http://bmjopen.bmj.com/site/about/guidelines.xhtml

#6	(#1 AND #3) OR (#2 AND #3) OR (#1 AND #3 AND #4) OR (#2 AND #3 AND #4) OR (#2 AND #5 AND #3) OR (#1 AND #2 AND #3) OR (#1 AND #2 AND #4 AND #3) OR (#1 AND #2 AND #5 AND #3)
#7	#6 NOT ("Animals"[Mesh] NOT "Humans"[Mesh])
#8	#7 AND "2001/01/01"[Date - Publication] : "2022/01/1"[Date - Publication]
#9	Systematic Review [Publication Type] OR "Review" [Publication Type]
#10	#8 NOT #9
#11	#10 AND (allchild[Filter])

Selection of Studies

The initial electronic database search will be performed to obtain all eligible records. Search results for each database will be exported to Endnote, where duplicates will be removed, and the number noted. At least two reviewers, independently, will screen the titles and abstracts for potential eligible studies and to exclude irrelevant studies using the Rayyan intelligent systematic reviews (RIS) tool. Disagreement will be resolved by discussion and consensus between the two reviewers or through the third reviewer contribution. Eligibility criteria will be based on the PECO approach, an acronym representing the population, exposure, comparator and the outcome [42]. P (Population) of interest are infants, children and adolescents, the E (exposure) is maternal cigarette smoking, maternal alcohol consumption, or both, the C (comparator) is the control group with no exposure to smoking and alcohol during pregnancy, and the O (outcomes) of interest are birth weight and cardio-metabolic risk factors. Thereafter, the full text will be retrieved and exported to Endnote and reviewed independently by two reviewers for a final selection of study into the review. Authors will be contacted if full texts cannot be retrieved. Experts in the field will be consulted to identify unpublished studies.

Studies will be selected if (i) relevant to the topic, (ii) study design is epidemiological observational studies such as prospective cohort studies, birth cohort studies and case-control studies, (iii) having exposure to maternal cigarette smoking or maternal alcohol consumption, (iv) having pregnant women as initially enrolled participants, (v) specified at least one adverse birth or child health outcome as an outcome of the investigation, (vi) investigated the association, correlation or causation (vii) having the largest sample size, if multiple studies used the same cohort. Only studies published in English will be included, however there will be no restriction regarding country, race, and gender. Studies focussing on mental health (including fetal alcohol syndrome), behavioural changes or cancer as the main outcome will be excluded.

For peer review only - http://bmjopen.bmj.com/site/about/guidelines.xhtml

Any disagreement about the inclusion of studies will be resolved through consultation of a third reviewer.

In addition to the database search, reference lists of selected articles will be examined to identify relevant studies that have not been captured in the database search. The flow chart will be used to summarize the search and selection of eligible studies. The PRISMA flow chart template will be used to summarize the search and selection of eligible studies [43]. Reasons of any exclusion of studies will be documented.

Quality and Risk of Bias Assessment

Quality assessment and risk of bias will be measured using the Newcastle Ottawa scale (NOS) (http://www.ohri.ca/programs/clinical_epidemiology/oxford.asp) for cohort and case-control studies. Cohort and case-control studies will be awarded a maximum score of nine stars. High quality studies will be defined as a quality score of seven or above and a quality score below seven will be deemed low quality.

Data Extraction and Management

All data extracted from the selected studies will be captured into a Microsoft excel document. The following data will be extracted from each selected study: authors, date of publication, country of data collection, population age at outcome assessment, sample size, study design, study setting, data collection method, type of exposure, source of data on maternal cigarette smoking/alcohol consumption, measure of the dependent variables, birth or child health outcomes, definition of outcome, statistical methods used to measure outcome, adjusted covariates and effect estimates (Appendix A). Effect estimates will include relative risks (95% CI) or adjusted odds ratios (OR's (95% CI)). Findings will include all birth outcomes or cardio-metabolic outcomes tested for an association with exposure to alcohol and cigarette smoking during pregnancy. Thereafter, the data will be extracted and stored on Review Manager (Revman Cochrane) for analyses and reporting. PRISMA guidelines will be used to summarize and report the review findings [43].

Data Synthesis and Analysis

Data Analysis

Findings from each study will be reported as a risk ratio or an odds ratio with the 95% confidence interval for dichotomous variables or mean difference with the standard deviation

For peer review only - http://bmjopen.bmj.com/site/about/guidelines.xhtml

for continuous variables. Studies with similar types of outcomes will be grouped to obtain feasible results on an overall estimate of effect. Due to the anticipated heterogeneity in included studies, a random effects meta-analysis will be considered.

Assessment of Heterogeneity

Heterogeneity will be evaluated through visual inspection of forest plots to judge the extent of Confidence Interval (CI) overlap. In addition, heterogeneity will be tested by the I^2 test. The I^2 statistic will classify heterogeneity. Heterogeneity among studies is expected due to risk factors defined differently according to age in children and adolescents. Therefore, the methods used to measure each health outcome are suspected to be different in children compared to adolescents. Additionally, heterogeneity may be due to characteristics such as gender, maternal health, maternal BMI and socioeconomic status. If the data allows, subgroup analysis will be performed. Similar risk factors will be grouped for comparison across studies. Risk factors according to age groups such as children or adolescents will be also be grouped.

Meta Analysis

Where there is sufficient reporting and a low degree of heterogeneity, a meta-analysis will be performed. Quantitative data will be pooled from individual studies to determine the pooled effect estimate with a fixed model preferred. A meta-analysis will be performed using Revman (Cochrane). After the meta-analysis, Grading of recommendations, assessment, development and evaluation (GRADEpro (Cochrane)) will be used to score and assess the overall certainty in the evidence for each outcome included in the analysis.

Subgroup Analysis

If the data allows, subgroup analysis will be performed. Similar risk factors will be grouped for comparison across studies. Risk factors according to age groups such as children or adolescents will also be grouped. Subgroup analysis will be performed on additional risk factors which may play a role in the adverse health outcomes (such as socioeconomic disadvantage, poor nutrition, second-hand smoke exposure, ethnicity, maternal stress, maternal mental health disorders, gestational diabetes, maternal BMI, poor dietary intake, physical inactivity, adolescent substance use and tobacco smoking). The pooled effect estimate will not be determined if the source of heterogeneity cannot be explained through subgroup analysis. In addition, if a meta-analysis cannot be performed due to few articles, insufficient reporting of data or high degree of heterogeneity, a narrative synthesis will be reported.

For peer review only - http://bmjopen.bmj.com/site/about/guidelines.xhtml

Publication Bias

Begg's funnel chart will be used to perform a visual inspection and evaluation of publication bias on the selected data. This is to exclude any publication bias.

Patient and Public Involvement

It was not appropriate or possible to involve patients or the public in the design, or conduct, or reporting, or dissemination plans of our research.

Ethics and dissemination

The review will extract publicly available secondary data and therefore do not require ethical review. The results will be written and submitted to a peer review journal.

Protocol registration

The systematic review protocol was registered with the international prospective register of systematic reviews (PROSPERO): Registration number: CRD42021286630. Details of this protocol can be accessed on PROSPERO (https://www.crd.york.ac.uk/prospero/display_record.php?RecordID=286630).

FUNDING

This research was made possible through funding by the Council for Scientific and Industrial Research (CSIR) (Grant number: NA).

AUTHOR CONTRIBUTIONS

TCH, JJADS and AO conceptualised and developed the systematic review protocol. TCH wrote the first draft of the protocol. ET edited the manuscript and developed the search strategy. Both TCH and ET will independently screen and select the title and abstracts of articles. Thereafter, the full-text will be retrieved for a final review and selection. Any discrepancies regarding the eligibility of articles will be resolved through consensus and, in the event of a disagreement regarding the inclusion of articles, a consultation with a third reviewer will be held. Reasons for the exclusion of studies will be reported. TCH will extract, analyse and synthesis the data under critical guidance of ET. All authors will critically revise the successive drafts of the manuscript and approve the final version.

Competing interests statement

The authors have no conflict of interest.

For peer review only - http://bmjopen.bmj.com/site/about/guidelines.xhtml

LIST OF ABBREVIATIONS

DOHaD	Developmental Origins of Health and Disease
CVD	Cardiovascular disease
MetS	Metabolic syndrome
BMI	Body Mass Index
SIDS	Sudden infant death syndrome
LBW	Low birth weight
IUGR	Intra-uterine growth restriction
SGA	Small-for-gestational age
HDL-cholesterol	High-density lipoprotein cholesterol
LDL cholesterol	Low-density lipoprotein cholesterol
cIMT	Carotid intima media thickness
aIMT	Aortic intima media thickness
PECO	Population, exposure, comparator, outcome
RIS tool	Rayyan intelligent systematic reviews tool
NOS	Newcastle Ottawa scale

For peer review only

For peer review only - http://bmjopen.bmj.com/site/about/guidelines.xhtml

REFERENCES

1 Dai X, Gakidou E, Lopez AD. Evolution of the global smoking epidemic over the past half century : strengthening the evidence base for policy action. 2022:129–37. doi:10.1136/tobaccocontrol-2021-056535

2 *Global status report on alcohol and health 2018*. Available from: https://www.who.int/publications-detail-redirect/9789241565639

3 Sciences H, Tavella RA, Muccillo-baisch ANAL. Prevalence of Illicit Drug Use During Pregnancy : A Global Perspective. 2020;**92**:1–18. doi:10.1590/0001-3765202020200302

4 Dukes K, Tripp T, Willinger M, *et al.* Drinking and smoking patterns during pregnancy: Development of group-based trajectories in the Safe Passage Study. *Alcohol* 2017;**62**:49–60. doi:10.1016/j.alcohol.2017.03.001

5 Maria C, Rodolfo P, Pacagnella C, *et al.* Drug Use during Pregnancy and its Consequences : A Nested Case Control Study on Severe Maternal Morbidity Uso de drogas na gestação e suas consequências : um estudo de caso-controle aninhado sobre morbidade materna grave. 2018;:518–26.

6 U.S Department of health and human services. *The Health Consequences of Smoking — 50 Years of Progress. A Report of the Surgeon General* 2014. Department of Health and Human Services, Centers for Disease Control and Prevention, National Center for Chronic Disease Prevention and Health Promotion, Office on Smoking and Health.

7 Dietz PM, England LJ, Shapiro-mendoza CK, *et al.* Infant Morbidity and Mortality Attributable to Prenatal Smoking in the U.S. *AMEPRE* 2010;**39**:45–52. doi:10.1016/j.amepre.2010.03.009

8 Kataria Y, Gaewsky L, Ellervik C. Prenatal smoking exposure and cardio-metabolic risk factors in adulthood : a general population study and a meta-analysis. *Int J Obes* 2018;**43**:763–73. doi:10.1038/s41366-018-0206-y

9 Leybovitz-Haleluya N, Wainstock T, Landau D, *et al.* Maternal smoking during pregnancy and the risk of pediatric cardiovascular diseases of the offspring: A population-based cohort study with up to 18-years of follow up. *Reprod Toxicol* 2018;**78**:69–74. doi:10.1016/j.reprotox.2018.03.009

10 Goodman E, Armstrong SC, Magge SN. The Metabolic Syndrome in Children and Adolescents : Shifting the Focus to Cardiometabolic Risk Factor Clustering. 2017;**140**.

11 Alfourti AMAB, Azzwali AAA, Azab AE. Effects of Maternal Nicotine Exposure during Pregnancy and Lactation on Blood Pressure of the Offspring and Blood Vessel Structure and Attenuation by Vitamin C. *East African Sch Journals Med Sci* 2019;**4421**.

12 Bakker H, Jaddoe VWV. Cardiovascular and metabolic influences of fetal smoke exposure. *Eur J Epidemiol* 2011;**26**:763–70. doi:10.1007/s10654-011-9621-2

13 Zhang CR, Kurniawan ND, Yamada L, *et al.* Early gestational ethanol exposure in mice: Effects on brain structure, energy metabolism and adiposity in adult offspring. *Alcohol* 2019;**75**:1–10. doi:10.1016/j.alcohol.2018.04.008

14 Akison LK, Reid N, Wyllie M, *et al.* Adverse health outcomes in offspring associated

For peer review only - http://bmjopen.bmj.com/site/about/guidelines.xhtml

with fetal alcohol exposure: A systematic review of clinical and preclinical studies with a focus on metabolic and body composition outcomes. *Alcohol Clin Exp Res* 2019;**43**:1324–43. doi:10.1111/acer.14078

15 Weeks O, Fazeli PK, Goessling W, *et al.* Fetal alcohol spectrum disorder predisposes to metabolic abnormalities in adulthood Fetal alcohol spectrum disorder predisposes to metabolic abnormalities in adulthood. 2020;**130**:2252–69.

16 Molnar DS, Rancourt D, Schlauch R, *et al.* Tobacco Exposure and Conditional Weight-for-Length Gain by 2 Years of Age. *J Pediatr Psychol* 2017;**42**:679–88. doi:10.1093/jpepsy/jsw095

17 Oken E, Huh SY, Taveras EM, *et al.* Associations of maternal prenatal smoking with child adiposity and blood pressure. *Obes Res* 2005;**13**:2021–8. doi:10.1038/oby.2005.248

18 Cupul-Uicab LA, Skjaerven R, Haug K, *et al.* In utero exposure to maternal tobacco smoke and subsequent obesity, hypertension, and gestational diabetes among women in the MoBa cohort. *Environ Health Perspect* 2012;**120**:355–60. doi:10.1289/ehp.1103789

19 World Health Organisation. Tobacco use and Second-Hand Smoke Exposure in Pregnancy. 2013. Available from: http://apps.who.int/iris/bitstream/handle/10665/94555/9789241506076_eng.pdf?sequence=1

20 Mandy M, Nyirenda M. Developmental Origins of Health and Disease: The relevance to developing nations. *Int Health* 2018;**10**:66–70. doi:10.1093/inthealth/ihy006

21 Kwok MK, Yeung SLA, Leung GM, *et al.* Birth weight and adult cardiovascular risk factors using multiple birth status as an instrumental variable in the 1958 British Birth Cohort. *Prev Med (Baltim)* 2016;**84**:69–75. doi:10.1016/j.ypmed.2015.12.016

22 Araújo CL, Hallal PC, Nader GA, *et al.* Effect of birth size and proportionality on BMI and skinfold thickness in early adolescence: Prospective birth cohort study. *Eur J Clin Nutr* 2009;**63**:634–9. doi:10.1038/ejcn.2008.20

23 Nordman H, Jääskeläinen J, Voutilainen R. Birth Size as a Determinant of Cardiometabolic Risk Factors in Children. 2020;:144–53. doi:10.1159/000509932

24 Finken MJJ, Steen M Van Der, Smeets CCJ, *et al.* Children Born Small for Gestational Age : 2018;:851–94. doi:10.1210/er.2018-00083

25 Tam CHT, Wang Y, Luan J, *et al.* Short Report : Epidemiology Non-linear relationship between birthweight and cardiometabolic risk factors in Chinese adolescents and adults. Published Online First: 2014. doi:10.1111/dme.12630

26 Koklu E, Kurtoglu S, Akcakus M, *et al.* Increased aortic intima-media thickness is related to lipid profile in newborns with intrauterine growth restriction. *Horm Res* 2006;**65**:269–75. doi:10.1159/000092536

27 De Smidt J, Odendaal HJ, Nel DG, *et al.* In utero teratogen exposure and cardiometabolic risk in 5-year-old children : a prospective pediatric study. *J Matern Neonatal Med* 2019;**0**:1–10. doi:10.1080/14767058.2019.1692337

28 Saenger P, Czernichow P, Hughes I, *et al.* Small for Gestational Age : Short Stature

For peer review only - http://bmjopen.bmj.com/site/about/guidelines.xhtml

and Beyond. 2007;**28**:219–51. doi:10.1210/er.2006-0039

29 Ponzio C, Palomino Z, Puccini RF. Does low birth weight affect the presence of cardiometabolic risk factors in overweight and obese children ? 2013;:1687–92. doi:10.1007/s00431-013-2113-5

30 Bruin JE, Gerstein HC, Holloway AC. Long-term consequences of fetal and neonatal nicotine exposure: A critical review. *Toxicol Sci* 2010;**116**:364–74. doi:10.1093/toxsci/kfq103

31 Cannon CP. Cardiovascular Disease and Modifiable Cardiometabolic Risk Factors. *Clin Cornerstone* 2007;**8**.

32 Davoudi-Kiakalayeh A, Mohammadi R, Pourfathollah AA, *et al.* Alloimmunization in thalassemia patients: New insight for healthcare. *Int J Prev Med* 2017;**8**:1–5. doi:10.4103/ijpvm.IJPVM

33 Myers B, Koen N, Donald KA, *et al.* Effect of Hazardous Alcohol Use During Pregnancy on Growth Outcomes at Birth: Findings from a South African Cohort Study. *Alcohol Clin Exp Res* 2018;**42**:369–77. doi:10.1111/acer.13566

34 Peltzer K, Pengpid S. Maternal alcohol use during pregnancy in a general national population in South Africa. *South African J Psychiatry* 2019;**25**:1–5. doi:10.4102/sajpsychiatry.v25i0.1236

35 Reid N, Akison LK, *et al.* Adverse Health Outcomes Associated With Fetal Alcohol Exposure : A Systematic Review Focused on Cardio – Renal Outcomes. 2010.

36 Oken E, Levitan EB, Gillman MW. Maternal smoking during pregnancy and child overweight : systematic review and meta-analysis. 2008;:201–10. doi:10.1038/sj.ijo.0803760

37 Rayfield S, Plugge E, Rayfield S, *et al.* Systematic review and meta-analysis of the association between maternal smoking in pregnancy and childhood overweight and obesity Stable URL : https://www.jstor.org/stable/26164426 Systematic review and meta-analysis of the association between maternal sm. 2016;**71**:162–73. doi:10.1136/jech-2016-207376

38 Sousa BA De, Horta BL. Maternal smoking during pregnancy and offspring body mass index and overweight : a systematic review and meta-analysis Tabagismo materno durante a gravidez e índice de massa corporal e excesso de peso entre os filhos : uma revisão sistemática e metanálise. 2019;**35**:1–18. doi:10.1590/0102-311X00176118

39 Seal N, Krakower G, Seal J. Maternal Smoking During Pregnancy and Metabolic Syndrome in Their Children. *TJNP J Nurse Pract* 2013;**9**:695–705. doi:10.1016/j.nurpra.2013.07.009

40 Shamseer L, Moher D, Ghersi D, *et al.* meta-analysis protocols (PRISMA-P) 2015 : elaboration. 2015;**7647**:1–25. doi:10.1136/bmj.g7647

41 Page MJ, Mckenzie JE, Bossuyt PM, *et al.* The PRISMA 2020 statement : an updated guideline for reporting systematic reviews Systematic reviews and Meta-Analyses. Published Online First: 2021. doi:10.1136/bmj.n71

42 Wickham J, Stehman S, Gass L, *et al.* Identifying the PECO: A framework for formulating good questions to explore the association of environmental and other

For peer review only

For peer review only - http://bmjopen.bmj.com/site/about/guidelines.xhtml

exposures with health outcomes. *Adv Ecol Res* 2019;**60**:1–24. doi:10.1016/j.envint.2018.07.015.Identifying

43 Moher D, Liberati A, Tetzlaff J, *et al.* Preferred Reporting Items for Systematic Reviews and Meta-Analyses : The PRISMA Statement. 2009;**6**. doi:10.1371/journal.pmed.1000097

For peer review only

For peer review only - http://bmjopen.bmj.com/site/about/guidelines.xhtml

Supplementary file 1:

Table 1: SCOPUS search strategy

Search #	Search terms TITLE-ABS-KEY
#1	TITLE-ABS-KEY (maternal smoking)
#2	"tobacco smoke expos*"
#3	"smoking during pregnancy"
#4	"prenatal smok*"
#5	smoking pregnant
#6	"cigarette smoking"
#7	"maternal smok*"
#8	#1 OR #2 OR #3 OR #4 OR #5 OR #6 OR #7
#9	TITLE-ABS-KEY (alcohol exposure)
#10	maternal alcohol exposure
#11	maternal alcohol consumption
#12	fetal alcohol*
#13	alcohol exposure in utero
#14	ethanol
#15	ethanol exposure
#16	#9 OR #10 OR #11 OR #12 OR #13 OR #14 OR #15
#17	TITLE-ABS-KEY (birth outcomes)
#18	birth defects
#19	Low-Birth-Weight
#20	LBW
#21	intrauterine growth restriction
#22	IUGR
#23	"Fetal Alcohol Spectrum Disorders"
#24	FASD
#25	#17 OR #18 OR #19 OR #20 OR #21 OR #22 OR #23 OR #24
#26	TITLE-ABS-KEY (Metabolic syndrome)
#27	"Syndrome X"
#28	cardiovascular risk*
#29	lipid*
#30	Total cholesterol

For peer review only

#31	Triglycerides
#32	abnormal lipid profile
#33	dyslipidaemia
#34	Diabet*
#35	blood glucose
#36	Hyperglyc*
#37	metabolic outcomes
#38	obesity
#39	body composition
#40	Body Mass Index
#41	BMI
#42	waist circumference
#43	Body Weight
#44	skinfold
#45	anthropometry
#46	anthropometric measurements
#47	adiposity
#48	blood pressure
#49	hypertens*
#50	#26 OR #27 OR #28 OR #29 OR #30 OR #31 OR #32 OR #33 OR #34 OR #35 OR #36 OR #37 OR #38 OR #39 OR #40 OR #41 OR #42 OR #43 OR #44 OR #45 OR #46 OR #47 OR #48 OR #49
#51	TITLE-ABS-KEY (intima media thickness)
#52	carotid intima media thickness
#53	aortic intima media thickness
#54	vascular dysfunction
#55	atherosclero*
#56	vascular stiffness
#57	arterial stiffness
#58	#51 OR #52 OR #53 OR #54 OR #55 OR #56 OR #57
#59	(#8 AND #25) OR (#16 AND #25) OR (#8 AND #25 AND #50) OR (#16 AND #25 AND #50) OR (#16 AND #25 AND #58) OR (#8 AND #16 AND #25) OR (#8 AND #16 AND #50 AND #25) OR (#8 AND #16 AND #25 AND #58)
#60	#56 AND (LIMIT-TO (PUBYEAR , 2001-2022))

For peer review only

Table 2: Web of Science search strategy

Search #	**Search terms**
#1	(TS= ((maternal smoking) OR ("tobacco smoke expos*") OR ("smoking during pregnancy") OR ("prenatal smok*") OR (smoke pregnant) OR (smoking pregnant) OR ("smoking during pregnancy") OR (cigarette smoking) OR ("maternal smok*")))
#2	(TS= ((alcohol exposure) OR (maternal alcohol exposure) OR (maternal alcohol consumption) OR (fetal alcohol) OR (foetal alcohol) OR (fetal alcohol exposure) OR (foetal exposure) OR (alcohol exposure in utero) OR (ethanol) OR (ethanol exposure) OR ("maternal alcohol expos*")))
#3	(TS= ((birth outcomes) OR (birth defects) OR (Low-Birth-Weight) OR (LBW) OR (intrauterine growth restriction) OR (IUGR) OR ("Fetal Alcohol Spectrum Disorders") OR (FASD)))
#4	(TS= ((Metabolic syndrome) OR ("Syndrome X") OR (cardiovascular risk) OR ("cardiometabolic risk*") OR (lipid*) OR (Total cholesterol) OR (Triglycerides) OR (abnormal lipid profile) OR (dyslipidemias) OR (dyslipidaemia) OR (diabetes) OR (diabetes mellitus) OR (blood glucose) OR (hyperglyc*) OR (metabolic outcomes) OR (obesity) OR (body composition) OR (Body Mass Index) OR (BMI) OR (waist circumference) OR (Body Weight) OR (skinfold) OR (anthropometry) OR (anthropometric measurements) OR (adiposity) OR (blood pressure) OR (hypertens*)))
#5	(TS= ((intima media thickness) OR (carotid intima media thickness) OR (aortic intima media thickness) OR (vascular dysfunction) OR (atherosclero*) OR (vascular stiffness) OR (arterial stiffness)))
#6	(#1 AND #3) OR (#2 AND #3) OR (#1 AND #3 AND #4) OR (#2 AND #3 AND #4) OR (#2 AND #5 AND #3) OR (#1 AND #2 AND #3) OR (#1 AND #2 AND #4 AND #3) OR (#1 AND #2 AND #5 AND #3)
#7	#6 AND (2001 OR 2002 OR 2003 OR 2004 OR 2005 OR 2006 OR 2007 OR 2008 OR 2009 OR 2010 OR 2011 OR 2012 OR 2013 OR 2014 OR 2015 OR 2016 OR 2017 OR 2018 OR 2019 OR 2020 OR 2021 OR 2022) PUBLICATION YEARS
#8	#7 AND Review Articles (Exclude – Document Types)
#9	#8 AND English (Languages)

For peer review only

APPENDIX A: DATA EXTRACTION FORM: Characteristics of studies

Authors	Year of publication	Country of data collection	Population age at outcome assessment	Sample size	Study design	Study setting	Data collection method

For peer review only

Type of exposure	Source of data on exposure	Measure of the dependent variables	Birth/child health outcomes	Definition of outcome	Statistical methods used to measure outcome	Study findings	Conclusion

For peer review only

Table 2| **PRISMA-P (preferred reporting items for systematic review and meta-analysis protocols) 2015 checklist: recommended items toaddress in a systematic review protocol**

Section and topic	Item No	Checklist item	Page & line number
**Administrative information**			
Title:			Page 1, line 1-3
Identification	1a	Identify the report as a protocol of a systematic review	Page 1, line 3
Update	1b	If the protocol is for an update of a previous systematic review, identify as such	N/A
Registration	2	If registered, provide the name of the registry (such as PROSPERO) and registration number	Page 2, line 34-35
Authors:			Page 1, line 5
Contact	3a	Provide name, institutional affiliation, e-mail address of all protocol authors; provide physical mailing address of corresponding author	Page 1, line 5-23
Contributions	3b	Describe contributions of protocol authors and identify the guarantor of the review	Page 13, line 18-27
Amendments	4	If the protocol represents an amendment of a previously completed or published protocol, identify as such and list changes; otherwise, state plan for documenting important protocol amendments	N/A
Support:			
Sources	5a	Indicate sources of financial or other support for the review	Page 13, line 15-17
Sponsor	5b	Provide name for the review funder and/or sponsor	Page 13, line 16-17
Role of sponsor or funder	5c	Describe roles of funder(s), sponsor(s), and/or institution(s), if any, in developing the protocol	
**Introduction**			
Rationale	6	Describe the rationale for the review in the context of what is already known	Page 4, line 1-page 6, line 15
Objectives	7	Provide an explicit statement of the question(s) the review will address with reference to participants, interventions, comparators, and outcomes (PICO)	Page 6, line 25- Page 7, line 4
**Methods**			
Eligibility criteria	8	Specify the study characteristics (such as PICO, study design, setting, time frame) and report characteristics (suchas years considered, language, publication status) to be used as criteria for eligibility for the review	Page 7, line 12-21 & Table 1
Information sources	9	Describe all intended information sources (such as electronic databases, contact with study authors, trial registersor other grey literature sources) with planned dates of coverage	Page 7, line 18-22
Search strategy	10	Present draft of search strategy to be used for at least one electronic database, including planned limits, such thatit could be repeated	Page 9, Table 2
Study records:			
Data management	11a	Describe the mechanism(s) that will be used to manage records and data throughout the review	Page 11, line 14-26
Selection process	11b	State the process that will be used for selecting studies (such as two independent reviewers) through each phaseof the review (that is, screening, eligibility and inclusion in meta-analysis)	Page 10, line 2-Page 11, line 2
Data collection process	11c	Describe planned method of extracting data from reports (such as piloting forms, done independently, in duplicate),any processes for obtaining and confirming data from investigators	Page 11, line 14-26
Data items	12	List and define all variables for which data will be sought (such as PICO items, funding sources), any pre-planneddata assumptions and simplifications	Page 11, line 30-page 12, line 4
Outcomes and prioritization	13	List and define all outcomes for which data will be sought, including prioritization of main and additional outcomes,with rationale	Page 8, line 8-16
Risk of bias in individual studies	14	Describe anticipated methods for assessing risk of bias of individual studies, including whether this will be done atthe outcome or study level, or both; state how this information will be used in data synthesis	Page 11, line 8-13
Data synthesis	15a	Describe criteria under which study data will be quantitatively synthesised	Page 11, line 28-page 12, line 1-3
	15b	If data are appropriate for quantitative synthesis, describe planned summary measures, methods of handling data and methods of combining data from studies, including any planned exploration of consistency (such as I2, Kendall's τ)	Page 12, line 4-20
	15c	Describe any proposed additional analyses (such as sensitivity or subgroup analyses, meta-regression)	Page 12, line 21-31
	15d	If quantitative synthesis is not appropriate, describe the type of summary planned	Page 12, line 30-31
Meta-bias(es)	16	Specify any planned assessment of meta-bias(es) (such as publication bias across studies, selective reporting within studies)	Page 13, line 1-3
Confidence in cumulative evidence	17	Describe how the strength of the body of evidence will be assessed (such as GRADE)	Page 12, line 18-20

For peer review only

BMJ Open

The effect of maternal cigarette smoking and alcohol consumption during pregnancy on birth weight and cardiometabolic risk factors in infants, children and adolescents: a systematic review protocol

Journal:	*BMJ Open*
Manuscript ID	bmjopen-2022-061811.R2
Article Type:	Protocol
Date Submitted by the Author:	15-Jun-2022
Complete List of Authors:	Hartel, Tammy; University of the Western Cape, Medical Bioscience<br>Turawa, Eunice; South African Medical Research Council, Burden of disease<br>Oelofse, Andre; University of the Western Cape, Medical Bioscience<br>De Smidt, Juley; University of the Western Cape, Medical Bioscience
**Primary Subject Heading**:	Paediatrics
Secondary Subject Heading:	Cardiovascular medicine, Research methods, Smoking and tobacco
Keywords:	Paediatric endocrinology < DIABETES & ENDOCRINOLOGY, Community child health < PAEDIATRICS, Maternal medicine < OBSTETRICS

SCHOLARONE™
Manuscripts

The effect of maternal cigarette smoking and alcohol consumption during pregnancy on birth weight and cardiometabolic risk factors in infants, children and adolescents: a systematic review protocol

Tammy C Hartel*[1], Eunice B Turawa[2], André Oelofse[1], Juléy JA De Smidt[1].

[1]Department of Medical Bioscience, Faculty of Natural Sciences, University of the Western Cape, Private Bag X17, Bellville, 7530, South Africa.

[2]Burden of Disease Unit, South African Medical Research Council, PO Box 19070, Tygerberg, 7505, South Africa.

*Correspondence to:

Tammy Hartel, Department of Medical Bioscience, Faculty of Natural Sciences, University of the Western Cape, Private Bag X17, Bellville, 7530, Cape Town, South Africa.

Tel: 082 778 1491

Email: 3366278@myuwc.ac.za

Co-Authors:

Eunice B Turawa

Email: Eunice.Turawa@mrc.ac.za

André Oelofse

Email: aoelofse@uwc.ac.za

Juléy JA De Smidt

Email: jdesmidt@uwc.ac.za

Word count: 3235

For peer review only - http://bmjopen.bmj.com/site/about/guidelines.xhtml

ABSTRACT

Introduction: Tobacco smoking and alcohol consumption during pregnancy are particularly prevalent in low socioeconomic status populations, with an adverse association with birth outcomes and cardiometabolic risk factors. However, the direct and indirect effects of prenatal cigarette smoking and alcohol consumption during pregnancy on cardiometabolic risk in offspring have been rather inconsistent. This may be attributed to multiple factors, such as the amount and timing of exposure to tobacco smoking and alcohol during pregnancy; the influence of maternal, environmental, and socioeconomic factors; or how risk factors were defined by individual researchers and studies. Therefore, this review aims to provide a summary of the most recent evidence on birth outcomes and cardiometabolic risk in children associated with alcohol and/or tobacco exposure *in utero*.

Methods and analysis: PubMed, Scopus, and Web of Science will be searched to identify published articles from Jan 1, 2001. Clinical studies that investigate the association between maternal cigarette smoking or alcohol consumption and birth weight and cardiometabolic risk factors in infants, children, and adolescents will be included. Prospective cohort, case-control studies, and birth cohort studies will be eligible for inclusion. Grey literature will be searched including conference proceedings, Google Scholar and ProQuest Dissertation and Theses database. Only studies published in English will be included, with no restrictions regarding country, race, or gender. Two independent reviewers will conduct the literature search and article screening. Eligibility criteria will be based on the population (infants, children, adolescents), exposure (maternal cigarette smoking, alcohol consumption, or both), comparator (control group with no exposure during pregnancy), and outcomes (birth weight and cardiometabolic risk factors). Quality assessment and risk of bias will be assessed using a risk of bias tool for observational studies, and data will be extracted for analysis using a researcher-generated data extraction form. A meta-analysis will be performed to estimate pooled effect sizes if there are sufficient good-quality studies available. Sources of heterogeneity will be explored using subgroup analysis.

Ethics and dissemination: Ethical clearance will not be required as this review will extract publicly available secondary data. Findings from this review will be disseminated via publication in a peer-review journal.

Study registration number: PROSPERO, CRD42021286630.

Keywords: Cardiometabolic risk factors, children, adolescents, alcohol exposure, maternal cigarette smoking, birth outcomes, systematic review.

For peer review only - http://bmjopen.bmj.com/site/about/guidelines.xhtml

Strengths and limitations of this study

- This review will synthesise literature from primary human studies that investigate the association, correlation, or causation between alcohol and/or tobacco exposure during pregnancy and birth weight or cardiometabolic risk factors in offspring.
- A comprehensive synthesis of all accessible data on outcomes of maternal cigarette smoking and/or alcohol consumption during pregnancy on birth weight and cardiometabolic risk factors in children will make use of a standardised risk of bias tool.
- The Grading of Recommendations Assessment Development and Evaluation (GRADE) will be used to assess the quality and strengthen the evidence from the review.
- Potential publication bias might limit the review; databases will therefore be searched to find unpublished studies such as thesis dissertations and conference proceedings to minimise the risk of publication bias.
- The review will be limited to evidence from an approximately two-decade period from 2001 to the final search.

For peer review only - http://bmjopen.bmj.com/site/about/guidelines.xhtml

INTRODUCTION

In 2020, the global prevalence of tobacco smoking among women was 6.5% [1]. This was attributed to a substantial decline in tobacco smoking of more than 40% in high socioeconomic countries over the past 50 years, however, there has been little to no decline in low to middle-income countries [1]. Globally, 32.4% of women 16 years and older were reported to be current alcohol consumers [2]. Despite the general decline in alcohol consumption and tobacco smoking among women globally, tobacco smoking remains a significant health burden, particularly among women of low socioeconomic status[1]. Women in low socioeconomic status communities were more likely to consume alcohol or use narcotics, concurrently with tobacco smoking during pregnancy, compared to women of higher socioeconomic status [3–5]. In addition, the global prevalence of illicit narcotic use during pregnancy was 1.6% in 2020, with studies reporting a 7.4 times higher prevalence after toxicological analysis [3].

In South Africa, 61.2% of women reported alcohol consumption, 56.3% reported smoking tobacco only, and 37.4% reported concomitant use of tobacco and alcohol during pregnancy[4]. Women of low education level, of low economic status, and those that had mental health disorders were more likely to smoke during pregnancy, relapse after pregnancy, and were less likely to quit smoking, compared to women with a higher education level and income status [6]. This, invariably, exacerbates the adverse effects on their offspring during and after pregnancy [6]. Similarly, biochemical analysis revealed that more than one in five pregnant women did not report their smoking status [7], resulting in underreported smoking and drinking patterns among pregnant women.

In the past decade, the research field of the Developmental Origins of Health and Disease (DOHaD) has significantly increased, with evidence growing on the detrimental effects of alcohol and tobacco exposure, *in-utero,* on the development of cardio-metabolic risk factors in adulthood [8,9]. Cardiometabolic risk factors include increased central adiposity, elevated triglycerides, decreased high-density lipoprotein cholesterol (HDL-cholesterol), elevated blood pressure, and hyperglycemia, predisposes individuals to developing type 2 diabetes mellitus and cardiovascular disease (CVD) [10]. Goodman, Armstrong and Magge, (2017) described Metabolic syndrome (MetS) as having at least three of these risk factors. Despite the challenges of defining metabolic syndrome in children and adolescents, Goodman, Armstrong and Magge (2017) emphasize that clinical screening should rather shift the focus to cardio-metabolic risk factors to address the major risks associated with metabolic syndrome. Moreover,

For peer review only - http://bmjopen.bmj.com/site/about/guidelines.xhtml

cardiometabolic risk factors were reported in offspring that were exposed to tobacco smoke or alcohol *in utero,* which needs further investigation [11–18]. Furthermore, infants born to mothers who smoke, or those that consume alcohol during pregnancy, were reported to have an increased risk of adverse birth outcomes, such as low birth weight [7,19]. This is augmented in infants born to mothers who smoked tobacco and consumed alcohol during pregnancy compared to those born to mothers that smoked only, consumed alcohol only, or abstained completely [20]. Therefore, birth weight is an important risk factor for cardiovascular disease and was shown to be significantly associated with BMI, skinfold thickness, and adolescent obesity [21,22], dyslipidaemia, altered glucose metabolism, hypertension, as well as low-grade inflammation [23].

Whether infants are born small or large for their gestational age, both have been reported to increase their risk of overweight or obesity later in life, due to catch-up growth [23–25]. Moreover, Koklu, Kurtoglu and Akcakuset et al (2006) reported a significantly higher aortic intima-media thickness in infants that were born small for gestational age, compared to infants born appropriate for their gestational age, that was also associated with hypertriglyceridemia [26]. Low birth weight, as well as small for gestational age was associated with higher carotid intima-media thickness values in children and linked to metabolic abnormalities, such as dyslipidemia, abdominal obesity, hypertension, and development of insulin resistance later in life [24,27,28]. Thus, overweight or obese children that were born with low birth weight are more likely to present with cardiometabolic risk factors, such as elevated systolic and diastolic blood pressures, triglycerides, and low-density lipoprotein cholesterol blood concentrations [29]. It is evident that tobacco smoking and alcohol use, during pregnancy, results in adverse birth outcomes which increases cardiometabolic health risks in both childhood and adulthood [30]. Therefore, the DOHaD may play a significant role in the development of cardiometabolic diseases in developing countries as poverty, malnutrition, licit and illicit narcotic use during pregnancy, as well as low birth weight are often prevalent in low socioeconomic regions [12].

In summary, to help prevent CVD later in life, it is important to assess adverse birth outcomes in infants and cardiometabolic risk factors in early childhood, including low birth weight, dyslipidaemia, abdominal obesity, hypertension, high blood glucose, and insulin resistance [31]. In South Africa, this is a concern as research has shown that tobacco smoking and alcohol consumption is highly prevalent in low socioeconomic populations while pregnant [4,20,32–34]. However, the evidence investigating the relationship between exposure to alcohol and tobacco during pregnancy and cardiometabolic outcomes in offspring is not consistent,

For peer review only - http://bmjopen.bmj.com/site/about/guidelines.xhtml

warranting the present review. Therefore, the direct and indirect effects of maternal cigarette smoking and/or alcohol consumption during pregnancy on cardiometabolic risk in offspring merits further investigation. This review aims to provide a summary of up-to-date evidence on the relationship between alcohol and/or tobacco exposure *in utero*, birth outcomes and cardio-metabolic outcomes in children.

Justification for this review

Previous systematic reviews have addressed cardio-renal outcomes, body composition, and metabolic outcomes in offspring exposed to alcohol during pregnancy [14,35], as well as maternal smoking during pregnancy and child diabetes mellitus type 2, whereas only a few systematic reviews addressed maternal smoking during pregnancy and childhood overweight and obesity [31,36–38]. Prenatal smoking and cardiometabolic risk factors were recently studied, but only among adults over 18 years of age [8]. Therefore, none of the latter has included all cardiometabolic risk factors in offspring and both maternal cigarette smoking and alcohol consumption during pregnancy. In addition, a review conducted in 2013 investigated maternal smoking during pregnancy and MetS in children and requires updating [39]. Therefore, a systematic review will be conducted to summarize the most recent evidence-based birth outcomes and cardiometabolic outcomes in children and adolescents in association with alcohol use and/or maternal cigarette smoking during pregnancy.

Aim, research question, and objectives

Aim

To provide up-to-date evidence-based birth outcomes and cardio-metabolic outcomes in children associated with maternal cigarette smoking only, maternal alcohol consumption only, or both maternal cigarette smoking and alcohol use during pregnancy.

Research question

What are the associated effects of cigarette smoking and/or alcohol consumption during pregnancy on birth weight, obesity, hypertension, diabetes, dyslipidaemia, and vascular dysfunction?

Objectives

Primary objective:

For peer review only - http://bmjopen.bmj.com/site/about/guidelines.xhtml

To assess birth weight and cardio-metabolic risk factors associated with maternal cigarette smoking and/or alcohol consumption during pregnancy.

Secondary objectives:

i. Identify potential mediators in the development of cardio-metabolic risk factors and vascular dysfunction in offspring exposed to alcohol consumption and/or cigarette smoking during pregnancy.
ii. Identify gaps in the literature and provide recommendations for future clinical studies.

METHODS AND ANALYSIS

Protocol reporting and registration

The methods for this review were developed according to the Preferred Reporting Items for Systematic Reviews and Meta-Analyses Protocols (PRISMA-P) and PRISMA 2020 statement [40,41]. The systematic review protocol was registered with the international prospective register of systematic reviews (PROSPERO): Registration number: CRD42021286630.

Criteria for consideration of studies

Types of Studies

Table 1 shows the list of inclusion and exclusion criteria. Types of studies will include human clinical studies that study the association, correlation, or causation between maternal cigarette smoking and alcohol consumption and cardio-metabolic risk factors in their children. Observational studies: Prospective studies (cohort studies, birth cohort studies, and prospective case-control studies) will be included. Data from primary population-based studies will be included only. Grey literature such as conference proceedings, ProQuest Dissertation and Theses database will be searched on the South African National Electronic Thesis and Dissertations (ETD) Portal and well as Google Scholar to reduce the risk of publication bias.

For peer review only - http://bmjopen.bmj.com/site/about/guidelines.xhtml

Table 1: Inclusion and exclusion criteria

Inclusion criteria	Exclusion criteria
• Studies that include pediatrics (Infants, children, adolescents).<br>• Observational studies: such as prospective cohort studies, birth cohort studies, and prospective case-control studies will be included.<br>• Grey literature: Conference proceedings, Ph.D. dissertations, and unpublished articles.<br>• Tobacco smoking in the form of cigarettes.<br>• Studies investigating the association, correlation, or causation between maternal cigarette smoking and/or alcohol consumption and cardiometabolic risk factors in offspring.<br>• Studies published after Jan 1, 2001.	• Studies that do not include infants, children, or adolescents.<br>• Studies that do not include at least one outcome of interest will be excluded.<br>• Cross-sectional studies, retrospective case-control studies, intervention studies, clinical trials, reviews and editorials, letters, and abstracts.<br>• Other forms of tobacco smoking such as electronic cigarettes, cigars, bidis, water pipes, and "I quit ordinary smoking" (IQOS) devices.

Types of participants

Pediatric patients are defined as:

i. Infants, children, and adolescents between the ages of 0 years to 19 years old.
ii. Infants, children, or adolescents exposed to maternal cigarette smoking only, maternal alcohol consumption only, or both maternal cigarette smoking and alcohol during pregnancy.

Main outcome measures

i. Birth outcomes: Low birth weight, birth weight.
ii. Anthropometry: Body Mass Index (BMI), Overweight, Obesity, Waist circumference.
iii. Blood pressure: Systolic blood pressure, Diastolic blood pressure, Hypertension, elevated blood pressure.
iv. Diabetes mellitus, Hyperglycemia.
v. Dyslipidaemia, Hypertriglyceridemia, High Low-density lipoprotein cholesterol, Low High-density lipoprotein cholesterol.
vi. Vascular dysfunction, increased intima-media thickness.

Search strategy

For peer review only - http://bmjopen.bmj.com/site/about/guidelines.xhtml

Using the comprehensive search terms, all relevant articles published, in English, from 2001, and indexed in PubMed, Google Scholar, Scopus, and Web of Science will be identified for inclusion. Relevant articles will be retrieved from 2001 to gather evidence over a two-decade period. The chosen period signalled the period when significant evidence-based on the current topic were published, we reckon that articles published before this period may not be relevant or of high quality to generate the robust evidence we are looking for in the current systematic review. The search strategies will incorporate both medical subject headings (MH) and free-text terms, and will be adapted to suit each database using applicable controlled vocabulary [Supplementary file 1]. An experienced information specialist will review the search terms to provide inputs and to ensure that search terms are relevant and optimally sensitive to identify eligible studies. An example of the search strategy in PubMed is displayed in table 2, and in Scopus and Web of Science as shown in supplementary tables 1 and 2, respectively. Each of the terms for exposure to maternal cigarette smoking or maternal alcohol consumption will be combined with each of the outcome terms described.

Electronic searches

Relevant studies will be searched for and retrieved from three databases: PubMed, Scopus, and Web of Science. In table 2, each of the terms for exposure to maternal cigarette smoking or alcohol consumption will be combined with each of the outcome terms described.

Table 2: Search strategy developed in PubMed

Search #	**Search terms**
#1	maternal smoking [tiab] OR maternal smok* OR intrauterine tobacco smoke expos* [tiab] OR maternal smoking during pregnancy [tiab] OR prenatal smoking [tiab] OR prenatal smoke expos* [tiab] OR smoke pregnant [tiab] OR smoking pregnant [tiab] smoking during pregnancy [tiab] OR cigarette smoking [tiab]
#2	alcohol exposure [tiab] OR maternal alcohol exposure [tiab] OR maternal alcohol expos* OR maternal alcohol consumption [tiab] OR maternal alcohol drinking OR fetal alcohol [tiab] OR foetal alcohol [tiab] OR fetal alcohol exposure [tiab] OR foetal exposure [tiab] OR alcohol exposure in utero [tiab] OR ethanol [MH] OR ethanol exposure [tiab]
#3	birth outcomes [tiab] OR birth defects [tiab] OR infant, Low Birth Weight [MH] OR birth weight [MH] OR birth length [tiab] OR LBW [tiab] OR intrauterine growth restriction [tiab] OR IUGR [tiab] OR Fetal Alcohol Spectrum Disorders [MH] OR FASD [tiab] OR FASDs [tiab]

For peer review only - http://bmjopen.bmj.com/site/about/guidelines.xhtml

#4	Metabolic syndrome [MH] OR Syndrome X [tiab] OR cardiovascular risk [tiab] OR cardiometabolic risk* [tiab] OR lipid profile [tiab] OR Lipids [MH] OR Total cholesterol [tiab] OR Triglycerides [MH] OR abnormal lipid profile [tiab] OR dyslipidemias [MH], OR dyslipidaemia [tiab] OR diabetes [tiab] OR diabetes mellitus [MH] OR blood glucose [MH] OR hyperglycemia [MH] OR hyperglycaemia [tiab] OR metabolic outcomes [tiab] OR obesity [MH] OR body composition [MH] OR Body Mass Index [MH] OR BMI [tiab] OR waist circumference [MH] OR Body Weights and Measures [MH] OR skinfold [tiab] OR anthropometry [MH] OR anthropometric measurements [tiab] OR adiposity [tiab] OR blood pressure [tiab] OR hypertens* [tiab]
#5	intima media thickness [tiab] OR carotid intima media thickness [MH] OR aortic intima media thickness [tiab] OR vascular dysfunction [tiab] OR atherosclerosis [MH] OR atherosclerotic lesions [tiab] OR Plaque, Atherosclerotic [MH] OR vascular stiffness [MH] OR arterial stiffness [tiab]
#6	(#1 AND #3) OR (#2 AND #3) OR (#1 AND #3 AND #4) OR (#2 AND #3 AND #4) OR (#2 AND #5 AND #3) OR (#1 AND #2 AND #3) OR (#1 AND #2 AND #4 AND #3) OR (#1 AND #2 AND #5 AND #3)
#7	#6 NOT ("Animals"[Mesh] NOT "Humans"[Mesh])
#8	#7 AND "2001/01/01"[Date - Publication] : "2022/01/1"[Date - Publication]
#9	Systematic Review [Publication Type] OR "Review" [Publication Type]
#10	#8 NOT #9
#11	#10 AND (allchild[Filter])

Selection of studies

The initial electronic database search will be performed to obtain all eligible records. Search results for each database will be exported to Endnote, where duplicates will be removed, and the number recorded. At least two reviewers will, independently, screen the titles and abstracts for potential eligible studies and exclude irrelevant studies using the Rayyan intelligent systematic reviews (RIS) tool. Disagreements will be resolved by discussion and consensus between the two reviewers or through the third reviewer's contribution. Eligibility criteria will be based on the PECO approach, an acronym representing the population, exposure, comparator, and outcome [42]. P (Population) of interest are infants, children, and adolescents, the E (exposure) is maternal cigarette smoking, maternal alcohol consumption, or both, and the C (comparator) is the control group with no exposure to smoking and alcohol during pregnancy, and the O (outcomes) of interest are birth weight and cardiometabolic risk factors. Thereafter, the full text will be retrieved and exported to Endnote and reviewed independently by the two

For peer review only - http://bmjopen.bmj.com/site/about/guidelines.xhtml

reviewers for a final selection of studies for inclusion into the review. Authors will be contacted if full texts cannot be retrieved. Experts in the field will be consulted to identify unpublished studies.

Studies will be selected if (ⅰ) relevant to the topic, (ⅱ) study design is epidemiological observational studies such as prospective cohort studies, birth cohort studies, and case-control studies, (ⅲ) having exposure to maternal cigarette smoking or maternal alcohol consumption, (ⅳ) having pregnant women as initially enrolled participants, (ⅴ) specified at least one adverse birth or child health outcome as an outcome of the investigation, (ⅵ) investigated the association, correlation or causation (ⅶ) having the largest sample size if multiple studies used the same cohort. Only studies published in English will be included, however, there will be no restrictions regarding country, race, and gender. Studies focussing on mental health, fetal alcohol syndrome, behavioural changes, or cancer as the main outcome will be excluded. Any disagreement about the inclusion of studies will be resolved through the consultation of a third reviewer.

In addition to the database search, reference lists of selected articles will be examined to identify relevant studies that have not been captured in the database search. The flow chart will be used to summarize the search and selection of eligible studies. The PRISMA flow chart template will be used to summarize the search and selection of eligible studies [43]. Reasons for any exclusion of studies will be documented.

Quality and risk of bias assessment

Quality assessment and risk of bias will be measured using the Newcastle Ottawa scale (NOS) (http://www.ohri.ca/programs/clinical_epidemiology/oxford.asp) for cohort and case-control studies. Cohort and case-control studies will be awarded a maximum score of nine stars. High-quality studies will be defined as a quality score of seven or above and a quality score below seven will be deemed low quality.

Data extraction and management

All data extracted from the selected studies will be captured into a Microsoft Excel document. The following data will be extracted from each selected study: authors, date of publication, country of data collection, population age at outcome assessment, sample size, study design, study setting, data collection method, type of exposure, source of data on maternal cigarette smoking/alcohol consumption, the measure of the dependent variables, birth or child health

For peer review only - http://bmjopen.bmj.com/site/about/guidelines.xhtml

outcomes, definition of the outcome, statistical methods used to measure the outcome, adjusted covariates and effect estimates (Appendix A). Effect estimates will include relative risks (95% CI) or adjusted odds ratios (OR's (95% CI)). Findings will include all birth outcomes or cardio-metabolic outcomes tested for an association with exposure to alcohol and cigarette smoking during pregnancy. Thereafter, the data will be extracted and stored on Review Manager (Revman Cochrane) for analyses and reporting. PRISMA guidelines will be used to summarize and report the review findings [43].

Data synthesis and analysis

Data analysis

Findings from each study will be reported as a risk ratio or an odds ratio with the 95% confidence interval for dichotomous variables or mean difference with the standard deviation for continuous variables. Studies with similar types of outcomes will be grouped to obtain feasible results on an overall estimate of effect. Due to the anticipated heterogeneity in included studies, a random-effects meta-analysis will be considered.

Assessment of heterogeneity

Heterogeneity will be evaluated through visual inspection of forest plots to judge the extent of Confidence Interval (CI) overlap. In addition, heterogeneity will be tested by the I^2 test. The I^2 statistic will classify heterogeneity. Heterogeneity among studies is expected due to risk factors defined differently according to age in children and adolescents. Therefore, the methods used to measure each health outcome are suspected to be different in children compared to adolescents. Additionally, heterogeneity may be due to characteristics such as gender, maternal health, maternal BMI, and socioeconomic status. If the data allows, subgroup analysis will be performed. Similar risk factors will be grouped for comparison across studies. Risk factors according to age groups such as children or adolescents will also be grouped.

Meta-analysis

Where there is sufficient reporting and a low degree of heterogeneity, a meta-analysis will be performed. Quantitative data will be pooled from individual studies to determine the pooled effect estimate with a fixed model preferred. A meta-analysis will be performed using Review Manager 5.3 (Cochrane). After the meta-analysis, the Grading of Recommendations, assessment, development, and evaluation (GRADEpro (Cochrane)) will be used to score and assess the overall certainty in the evidence for each outcome included in the analysis.

For peer review only - http://bmjopen.bmj.com/site/about/guidelines.xhtml

Subgroup analysis

If the data allows, subgroup analysis will be performed. Similar risk factors will be grouped for comparison across studies. Risk factors according to age groups such as children or adolescents will also be grouped. Subgroup analysis will be performed on additional risk factors which may play a role in the adverse health outcomes (such as socioeconomic disadvantage, poor nutrition, second-hand smoke exposure, ethnicity, maternal stress, maternal mental health disorders, gestational diabetes, maternal BMI, poor dietary intake, physical inactivity, adolescent substance use, and tobacco smoking). The pooled effect estimate will not be determined if the source of heterogeneity cannot be explained through subgroup analysis. In addition, if a meta-analysis cannot be performed due to few articles, insufficient reporting of data, or a high degree of heterogeneity, a narrative synthesis will be reported.

Publication bias

Begg's funnel chart will be used to perform a visual inspection and evaluation of publication bias on the selected data. This is to exclude any publication bias.

Patient and public involvement

It was not appropriate or possible to involve patients or the public in the design, conduct, reporting, or dissemination plans of our research.

ETHICS AND DISSEMINATION

The review will extract publicly available secondary data and therefore does not require ethical review. The results will be submitted for publication in a peer-review journal.

For peer review only - http://bmjopen.bmj.com/site/about/guidelines.xhtml

Contributors

TCH, JJADS, and AO conceptualised and developed the systematic review protocol. TCH wrote the first draft of the protocol. ET edited the manuscript and developed the search strategy. Both TCH and ET will independently screen and select the title and abstracts of articles. Thereafter, the full text will be retrieved for a final review and selection. Any discrepancies regarding the eligibility of articles will be resolved through consensus and, in the event of a disagreement regarding the inclusion of articles, a consultation with a third reviewer will be held. Reasons for the exclusion of studies will be reported. TCH will extract, analyse, and synthesis the data under the critical guidance of ET. All authors will critically revise the successive drafts of the manuscript and approve the final version.

Funding

This research was made possible through funding by the Council for Scientific and Industrial Research (CSIR) (Grant number: NA).

Competing interests

The authors have no conflicts of interest.

For peer review only - http://bmjopen.bmj.com/site/about/guidelines.xhtml

LIST OF ABBREVIATIONS

DOHaD	Developmental Origins of Health and Disease
CVD	Cardiovascular disease
MetS	Metabolic syndrome
BMI	Body Mass Index
SIDS	Sudden infant death syndrome
LBW	Low birth weight
IUGR	Intra-uterine growth restriction
SGA	Small-for-gestational age
HDL-cholesterol	High-density lipoprotein cholesterol
LDL cholesterol	Low-density lipoprotein cholesterol
cIMT	Carotid intima media thickness
aIMT	Aortic intima media thickness
PECO	Population, exposure, comparator, outcome
RIS tool	Rayyan intelligent systematic reviews tool
NOS	Newcastle Ottawa scale

For peer review only

For peer review only - http://bmjopen.bmj.com/site/about/guidelines.xhtml

References

1 Dai X, Gakidou E, Lopez AD. Evolution of the global smoking epidemic over the past half century : strengthening the evidence base for policy action. 2022:129–37. doi:10.1136/tobaccocontrol-2021-056535

2 *Global status report on alcohol and health 2018*. Available from: https://www.who.int/publications-detail-redirect/9789241565639

3 Sciences H, Tavella RA, Muccillo-baisch ANAL. Prevalence of Illicit Drug Use During Pregnancy : A Global Perspective. 2020;**92**:1–18. doi:10.1590/0001-3765202020200302

4 Dukes K, Tripp T, Willinger M, *et al.* Drinking and smoking patterns during pregnancy: Development of group-based trajectories in the Safe Passage Study. *Alcohol* 2017;**62**:49–60. doi:10.1016/j.alcohol.2017.03.001

5 Maria C, Rodolfo P, Pacagnella C, *et al.* Drug Use during Pregnancy and its Consequences : A Nested Case Control Study on Severe Maternal Morbidity Uso de drogas na gestação e suas consequências : um estudo de caso-controle aninhado sobre morbidade materna grave. 2018;:518–26.

6 U.S Department of health and human services. *The Health Consequences of Smoking — 50 Years of Progress. A Report of the Surgeon General* 2014. Department of Health and Human Services, Centers for Disease Control and Prevention, National Center for Chronic Disease Prevention and Health Promotion, Office on Smoking and Health.

7 Dietz PM, England LJ, Shapiro-mendoza CK, *et al.* Infant Morbidity and Mortality Attributable to Prenatal Smoking in the U.S. *AMEPRE* 2010;**39**:45–52. doi:10.1016/j.amepre.2010.03.009

8 Kataria Y, Gaewsky L, Ellervik C. Prenatal smoking exposure and cardio-metabolic risk factors in adulthood : a general population study and a meta-analysis. *Int J Obes* 2018;**43**:763–73. doi:10.1038/s41366-018-0206-y

9 Leybovitz-Haleluya N, Wainstock T, Landau D, *et al.* Maternal smoking during pregnancy and the risk of pediatric cardiovascular diseases of the offspring: A population-based cohort study with up to 18-years of follow up. *Reprod Toxicol* 2018;**78**:69–74. doi:10.1016/j.reprotox.2018.03.009

10 Goodman E, Armstrong SC, Magge SN. The Metabolic Syndrome in Children and Adolescents : Shifting the Focus to Cardiometabolic Risk Factor Clustering. 2017;**140**.

11 Alfourti AMAB, Azzwali AAA, Azab AE. Effects of Maternal Nicotine Exposure during Pregnancy and Lactation on Blood Pressure of the Offspring and Blood Vessel Structure and Attenuation by Vitamin C. *East African Sch Journals Med Sci* 2019;**4421**.

12 Bakker H, Jaddoe VWV. Cardiovascular and metabolic influences of fetal smoke exposure. *Eur J Epidemiol* 2011;**26**:763–70. doi:10.1007/s10654-011-9621-2

13 Zhang CR, Kurniawan ND, Yamada L, *et al.* Early gestational ethanol exposure in mice: Effects on brain structure, energy metabolism and adiposity in adult offspring. *Alcohol* 2019;**75**:1–10. doi:10.1016/j.alcohol.2018.04.008

14 Akison LK, Reid N, Wyllie M, *et al.* Adverse health outcomes in offspring associated

For peer review only - http://bmjopen.bmj.com/site/about/guidelines.xhtml

with fetal alcohol exposure: A systematic review of clinical and preclinical studies with a focus on metabolic and body composition outcomes. *Alcohol Clin Exp Res* 2019;**43**:1324–43. doi:10.1111/acer.14078

15 Weeks O, Fazeli PK, Goessling W, *et al.* Fetal alcohol spectrum disorder predisposes to metabolic abnormalities in adulthood Fetal alcohol spectrum disorder predisposes to metabolic abnormalities in adulthood. 2020;**130**:2252–69.

16 Molnar DS, Rancourt D, Schlauch R, *et al.* Tobacco Exposure and Conditional Weight-for-Length Gain by 2 Years of Age. *J Pediatr Psychol* 2017;**42**:679–88. doi:10.1093/jpepsy/jsw095

17 Oken E, Huh SY, Taveras EM, *et al.* Associations of maternal prenatal smoking with child adiposity and blood pressure. *Obes Res* 2005;**13**:2021–8. doi:10.1038/oby.2005.248

18 Cupul-Uicab LA, Skjaerven R, Haug K, *et al.* In utero exposure to maternal tobacco smoke and subsequent obesity, hypertension, and gestational diabetes among women in the MoBa cohort. *Environ Health Perspect* 2012;**120**:355–60. doi:10.1289/ehp.1103789

19 World Health Organisation. Tobacco use and Second-Hand Smoke Exposure in Pregnancy. 2013. Available from: http://apps.who.int/iris/bitstream/handle/10665/94555/9789241506076_eng.pdf?sequence=1

20 Mandy M, Nyirenda M. Developmental Origins of Health and Disease: The relevance to developing nations. *Int Health* 2018;**10**:66–70. doi:10.1093/inthealth/ihy006

21 Kwok MK, Yeung SLA, Leung GM, *et al.* Birth weight and adult cardiovascular risk factors using multiple birth status as an instrumental variable in the 1958 British Birth Cohort. *Prev Med (Baltim)* 2016;**84**:69–75. doi:10.1016/j.ypmed.2015.12.016

22 Araújo CL, Hallal PC, Nader GA, *et al.* Effect of birth size and proportionality on BMI and skinfold thickness in early adolescence: Prospective birth cohort study. *Eur J Clin Nutr* 2009;**63**:634–9. doi:10.1038/ejcn.2008.20

23 Nordman H, Jääskeläinen J, Voutilainen R. Birth Size as a Determinant of Cardiometabolic Risk Factors in Children. 2020;:144–53. doi:10.1159/000509932

24 Finken MJJ, Steen M Van Der, Smeets CCJ, *et al.* Children Born Small for Gestational Age : 2018;:851–94. doi:10.1210/er.2018-00083

25 Tam CHT, Wang Y, Luan J, *et al.* Short Report : Epidemiology Non-linear relationship between birthweight and cardiometabolic risk factors in Chinese adolescents and adults. Published Online First: 2014. doi:10.1111/dme.12630

26 Koklu E, Kurtoglu S, Akcakus M, *et al.* Increased aortic intima-media thickness is related to lipid profile in newborns with intrauterine growth restriction. *Horm Res* 2006;**65**:269–75. doi:10.1159/000092536

27 De Smidt J, Odendaal HJ, Nel DG, *et al.* In utero teratogen exposure and cardiometabolic risk in 5-year-old children : a prospective pediatric study. *J Matern Neonatal Med* 2019;**0**:1–10. doi:10.1080/14767058.2019.1692337

28 Saenger P, Czernichow P, Hughes I, *et al.* Small for Gestational Age : Short Stature

For peer review only - http://bmjopen.bmj.com/site/about/guidelines.xhtml

and Beyond. 2007;**28**:219–51. doi:10.1210/er.2006-0039

29 Ponzio C, Palomino Z, Puccini RF. Does low birth weight affect the presence of cardiometabolic risk factors in overweight and obese children ? 2013;:1687–92. doi:10.1007/s00431-013-2113-5

30 Bruin JE, Gerstein HC, Holloway AC. Long-term consequences of fetal and neonatal nicotine exposure: A critical review. *Toxicol Sci* 2010;**116**:364–74. doi:10.1093/toxsci/kfq103

31 Cannon CP. Cardiovascular Disease and Modifiable Cardiometabolic Risk Factors. *Clin Cornerstone* 2007;**8**.

32 Davoudi-Kiakalayeh A, Mohammadi R, Pourfathollah AA, *et al.* Alloimmunization in thalassemia patients: New insight for healthcare. *Int J Prev Med* 2017;**8**:1–5. doi:10.4103/ijpvm.IJPVM

33 Myers B, Koen N, Donald KA, *et al.* Effect of Hazardous Alcohol Use During Pregnancy on Growth Outcomes at Birth: Findings from a South African Cohort Study. *Alcohol Clin Exp Res* 2018;**42**:369–77. doi:10.1111/acer.13566

34 Peltzer K, Pengpid S. Maternal alcohol use during pregnancy in a general national population in South Africa. *South African J Psychiatry* 2019;**25**:1–5. doi:10.4102/sajpsychiatry.v25i0.1236

35 Reid N, Akison LK, *et al.* Adverse Health Outcomes Associated With Fetal Alcohol Exposure : A Systematic Review Focused on Cardio – Renal Outcomes. 2010.

36 Oken E, Levitan EB, Gillman MW. Maternal smoking during pregnancy and child overweight : systematic review and meta-analysis. 2008;:201–10. doi:10.1038/sj.ijo.0803760

37 Rayfield S, Plugge E, Rayfield S, *et al.* Systematic review and meta-analysis of the association between maternal smoking in pregnancy and childhood overweight and obesity Stable URL : https://www.jstor.org/stable/26164426 Systematic review and meta-analysis of the association between maternal sm. 2016;**71**:162–73. doi:10.1136/jech-2016-207376

38 Sousa BA De, Horta BL. Maternal smoking during pregnancy and offspring body mass index and overweight : a systematic review and meta-analysis Tabagismo materno durante a gravidez e índice de massa corporal e excesso de peso entre os filhos : uma revisão sistemática e metanálise. 2019;**35**:1–18. doi:10.1590/0102-311X00176118

39 Seal N, Krakower G, Seal J. Maternal Smoking During Pregnancy and Metabolic Syndrome in Their Children. *TJNP J Nurse Pract* 2013;**9**:695–705. doi:10.1016/j.nurpra.2013.07.009

40 Shamseer L, Moher D, Ghersi D, *et al.* meta-analysis protocols (PRISMA-P) 2015 : elaboration. 2015;**7647**:1–25. doi:10.1136/bmj.g7647

41 Page MJ, Mckenzie JE, Bossuyt PM, *et al.* The PRISMA 2020 statement : an updated guideline for reporting systematic reviews Systematic reviews and Meta-Analyses. Published Online First: 2021. doi:10.1136/bmj.n71

42 Wickham J, Stehman S, Gass L, *et al.* Identifying the PECO: A framework for formulating good questions to explore the association of environmental and other

For peer review only

For peer review only - http://bmjopen.bmj.com/site/about/guidelines.xhtml

exposures with health outcomes. *Adv Ecol Res* 2019;**60**:1–24. doi:10.1016/j.envint.2018.07.015.Identifying

43 Moher D, Liberati A, Tetzlaff J, *et al.* Preferred Reporting Items for Systematic Reviews and Meta-Analyses : The PRISMA Statement. 2009;**6**. doi:10.1371/journal.pmed.1000097

For peer review only

For peer review only - http://bmjopen.bmj.com/site/about/guidelines.xhtml

APPENDIX A: DATA EXTRACTION FORM: Characteristics of studies

Authors	Year of publication	Country of data collection	Population age at outcome assessment	Sample size	Study design	Study setting	Data collection method

For peer review only

Type of exposure	Source of data on exposure	Measure of the dependent variables	Birth/child health outcomes	Definition of outcome	Statistical methods used to measure outcome	Study findings	Conclusion

For peer review only

Supplementary file 1:

Table 1: SCOPUS search strategy

Search #	Search terms TITLE-ABS-KEY
#1	TITLE-ABS-KEY (maternal smoking)
#2	"tobacco smoke expos*"
#3	"smoking during pregnancy"
#4	"prenatal smok*"
#5	smoking pregnant
#6	"cigarette smoking"
#7	"maternal smok*"
#8	#1 OR #2 OR #3 OR #4 OR #5 OR #6 OR #7
#9	TITLE-ABS-KEY (alcohol exposure)
#10	maternal alcohol exposure
#11	maternal alcohol consumption
#12	fetal alcohol*
#13	alcohol exposure in utero
#14	ethanol
#15	ethanol exposure
#16	#9 OR #10 OR #11 OR #12 OR #13 OR #14 OR #15
#17	TITLE-ABS-KEY (birth outcomes)
#18	birth defects
#19	Low-Birth-Weight
#20	LBW
#21	intrauterine growth restriction
#22	IUGR
#23	"Fetal Alcohol Spectrum Disorders"
#24	FASD
#25	#17 OR #18 OR #19 OR #20 OR #21 OR #22 OR #23 OR #24
#26	TITLE-ABS-KEY (Metabolic syndrome)
#27	"Syndrome X"
#28	cardiovascular risk*
#29	lipid*
#30	Total cholesterol

For peer review only

#31	Triglycerides
#32	abnormal lipid profile
#33	dyslipidaemia
#34	Diabet*
#35	blood glucose
#36	Hyperglyc*
#37	metabolic outcomes
#38	obesity
#39	body composition
#40	Body Mass Index
#41	BMI
#42	waist circumference
#43	Body Weight
#44	skinfold
#45	anthropometry
#46	anthropometric measurements
#47	adiposity
#48	blood pressure
#49	hypertens*
#50	#26 OR #27 OR #28 OR #29 OR #30 OR #31 OR #32 OR #33 OR #34 OR #35 OR #36 OR #37 OR #38 OR #39 OR #40 OR #41 OR #42 OR #43 OR #44 OR #45 OR #46 OR #47 OR #48 OR #49
#51	TITLE-ABS-KEY (intima media thickness)
#52	carotid intima media thickness
#53	aortic intima media thickness
#54	vascular dysfunction
#55	atherosclero*
#56	vascular stiffness
#57	arterial stiffness
#58	#51 OR #52 OR #53 OR #54 OR #55 OR #56 OR #57
#59	(#8 AND #25) OR (#16 AND #25) OR (#8 AND #25 AND #50) OR (#16 AND #25 AND #50) OR (#16 AND #25 AND #58) OR (#8 AND #16 AND #25) OR (#8 AND #16 AND #50 AND #25) OR (#8 AND #16 AND #25 AND #58)
#60	#56 AND (LIMIT-TO (PUBYEAR , 2001-2022))

For peer review only

Table 2: Web of Science search strategy

Search #	Search terms
#1	(TS= ((maternal smoking) OR ("tobacco smoke expos*") OR ("smoking during pregnancy") OR ("prenatal smok*") OR (smoke pregnant) OR (smoking pregnant) OR ("smoking during pregnancy") OR (cigarette smoking) OR ("maternal smok*")))
#2	(TS= ((alcohol exposure) OR (maternal alcohol exposure) OR (maternal alcohol consumption) OR (fetal alcohol) OR (foetal alcohol) OR (fetal alcohol exposure) OR (foetal exposure) OR (alcohol exposure in utero) OR (ethanol) OR (ethanol exposure) OR ("maternal alcohol expos*")))
#3	(TS= ((birth outcomes) OR (birth defects) OR (Low-Birth-Weight) OR (LBW) OR (intrauterine growth restriction) OR (IUGR) OR ("Fetal Alcohol Spectrum Disorders") OR (FASD)))
#4	(TS= ((Metabolic syndrome) OR ("Syndrome X") OR (cardiovascular risk) OR ("cardiometabolic risk*") OR (lipid*) OR (Total cholesterol) OR (Triglycerides) OR (abnormal lipid profile) OR (dyslipidemias) OR (dyslipidaemia) OR (diabetes) OR (diabetes mellitus) OR (blood glucose) OR (hyperglyc*) OR (metabolic outcomes) OR (obesity) OR (body composition) OR (Body Mass Index) OR (BMI) OR (waist circumference) OR (Body Weight) OR (skinfold) OR (anthropometry) OR (anthropometric measurements) OR (adiposity) OR (blood pressure) OR (hypertens*)))
#5	(TS= ((intima media thickness) OR (carotid intima media thickness) OR (aortic intima media thickness) OR (vascular dysfunction) OR (atherosclero*) OR (vascular stiffness) OR (arterial stiffness)))
#6	(#1 AND #3) OR (#2 AND #3) OR (#1 AND #3 AND #4) OR (#2 AND #3 AND #4) OR (#2 AND #5 AND #3) OR (#1 AND #2 AND #3) OR (#1 AND #2 AND #4 AND #3) OR (#1 AND #2 AND #5 AND #3)
#7	#6 AND (2001 OR 2002 OR 2003 OR 2004 OR 2005 OR 2006 OR 2007 OR 2008 OR 2009 OR 2010 OR 2011 OR 2012 OR 2013 OR 2014 OR 2015 OR 2016 OR 2017 OR 2018 OR 2019 OR 2020 OR 2021 OR 2022) PUBLICATION YEARS
#8	#7 AND Review Articles (Exclude – Document Types)
#9	#8 AND English (Languages)

For peer review only

Table 2| **PRISMA-P (preferred reporting items for systematic review and meta-analysis protocols) 2015 checklist: recommended items toaddress in a systematic review protocol**

Section and topic	Item No	Checklist item	Page & line number
**Administrative information**			
Title:			Page 1, line 1-3
Identification	1a	Identify the report as a protocol of a systematic review	Page 1, line 3
Update	1b	If the protocol is for an update of a previous systematic review, identify as such	N/A
Registration	2	If registered, provide the name of the registry (such as PROSPERO) and registration number	Page 2, line 34-35
Authors:			Page 1, line 5
Contact	3a	Provide name, institutional affiliation, e-mail address of all protocol authors; provide physical mailing address of corresponding author	Page 1, line 5-23
Contributions	3b	Describe contributions of protocol authors and identify the guarantor of the review	Page 13, line 18-27
Amendments	4	If the protocol represents an amendment of a previously completed or published protocol, identify as such and list changes; otherwise, state plan for documenting important protocol amendments	N/A
Support:			
Sources	5a	Indicate sources of financial or other support for the review	Page 13, line 15-17
Sponsor	5b	Provide name for the review funder and/or sponsor	Page 13, line 16-17
Role of sponsor or funder	5c	Describe roles of funder(s), sponsor(s), and/or institution(s), if any, in developing the protocol	
**Introduction**			
Rationale	6	Describe the rationale for the review in the context of what is already known	Page 4, line 1-page 6, line 15
Objectives	7	Provide an explicit statement of the question(s) the review will address with reference to participants, interventions, comparators, and outcomes (PICO)	Page 6, line 25- Page 7, line 4
**Methods**			
Eligibility criteria	8	Specify the study characteristics (such as PICO, study design, setting, time frame) and report characteristics (suchas years considered, language, publication status) to be used as criteria for eligibility for the review	Page 7, line 12-21 & Table 1
Information sources	9	Describe all intended information sources (such as electronic databases, contact with study authors, trial registersor other grey literature sources) with planned dates of coverage	Page 7, line 18-22
Search strategy	10	Present draft of search strategy to be used for at least one electronic database, including planned limits, such thatit could be repeated	Page 9, Table 2
Study records:			
Data management	11a	Describe the mechanism(s) that will be used to manage records and data throughout the review	Page 11, line 14-26
Selection process	11b	State the process that will be used for selecting studies (such as two independent reviewers) through each phaseof the review (that is, screening, eligibility and inclusion in meta-analysis)	Page 10, line 2-Page 11, line 2
Data collection process	11c	Describe planned method of extracting data from reports (such as piloting forms, done independently, in duplicate),any processes for obtaining and confirming data from investigators	Page 11, line 14-26
Data items	12	List and define all variables for which data will be sought (such as PICO items, funding sources), any pre-planneddata assumptions and simplifications	Page 11, line 30-page 12, line 4
Outcomes and prioritization	13	List and define all outcomes for which data will be sought, including prioritization of main and additional outcomes,with rationale	Page 8, line 8-16
Risk of bias in individual studies	14	Describe anticipated methods for assessing risk of bias of individual studies, including whether this will be done atthe outcome or study level, or both; state how this information will be used in data synthesis	Page 11, line 8-13
Data synthesis	15a	Describe criteria under which study data will be quantitatively synthesised	Page 11, line 28-page 12, line 1-3
	15b	If data are appropriate for quantitative synthesis, describe planned summary measures, methods of handling data and methods of combining data from studies, including any planned exploration of consistency (such as I2, Kendall's τ)	Page 12, line 4-20
	15c	Describe any proposed additional analyses (such as sensitivity or subgroup analyses, meta-regression)	Page 12, line 21-31
	15d	If quantitative synthesis is not appropriate, describe the type of summary planned	Page 12, line 30-31
Meta-bias(es)	16	Specify any planned assessment of meta-bias(es) (such as publication bias across studies, selective reporting within studies)	Page 13, line 1-3
Confidence in cumulative evidence	17	Describe how the strength of the body of evidence will be assessed (such as GRADE)	Page 12, line 18-20

For peer review only
